# Epigenetic alterations in cancer metastasis: molecular mechanisms and implications for precision oncology

**DOI:** 10.3389/fonc.2026.1788808

**Published:** 2026-04-29

**Authors:** Seval Turna, Semra Demokan

**Affiliations:** 1 Institute of Graduate Studies in Health Sciences, Istanbul University, Istanbul, Türkiye; 2 School of Health Services Vocational College, Pathology Laboratory Techniques Program, Bezmialem Vakif University, Istanbul, Türkiye; 3 Department of Basic Oncology, Experimental and Molecular Oncology Division, Oncology Institute, Istanbul University, Istanbul, Türkiye

**Keywords:** cancer metastasis, metastatic cascade, epigenetic regulation, epithelial-mesenchymal transition, DNA methylation, histone modifications, and precision oncology

## Abstract

Metastasis is a complex, multistep process that underlies the majority of cancer-related deaths and is increasingly recognized as a consequence of dynamic epigenetic reprogramming rather than solely genetic alterations. Epigenetic mechanisms enable stable yet reversible changes in gene expression that drive cellular plasticity throughout the metastatic cascade, including epithelial–mesenchymal transition (EMT), intravasation, survival as circulating tumor cells, extravasation, dormancy, and metastatic colonization. In this review, we provide an integrated overview of the molecular epigenetic mechanisms regulating cancer metastasis, with particular emphasis on DNA methylation, histone post-translational modifications, chromatin remodeling complexes, and non-coding RNAs. We discuss how these epigenetic regulators interact with key EMT-associated signaling pathways to control phenotypic plasticity, anoikis resistance, and metastatic niche adaptation. In addition, emerging evidence supports the clinical relevance of metastasis-associated epigenetic alterations, including DNA methylation signatures, histone modification patterns, and non-coding RNA profiles, as promising diagnostic and prognostic biomarkers. Selected examples such as aberrant CDH1 promoter methylation, miR-200 family dysregulation, and EZH2-mediated chromatin remodeling illustrate their potential utility in patient stratification and therapeutic targeting. A deeper mechanistic understanding of epigenetic regulation across metastatic stages may facilitate the development of clinically actionable biomarkers and combination therapies in precision oncology.

## Introduction

Metastasis, a defining hallmark of cancer, refers to the dissemination of malignant cells from the primary tumor to distant anatomical sites, where they establish secondary lesions (1–3). Despite advances in early detection and localized treatment, metastasis remains responsible for the vast majority of cancer-related deaths. Remarkably, metastatic spread can be initiated by a very small subpopulation of tumor cells—estimated to be as few as 0.01% of primary tumor cells—that successfully intravasate, survive within the circulation, and colonize distant organs (1, 3, 4). While genetic alterations contribute to clonal selection and tumor evolution, accumulating evidence indicates that epigenetic plasticity, rather than fixed genetic mutations alone, serves as a central and dynamic driver of metastatic progression (5–7).

Metastasis is not a single event, but a highly orchestrated, multistep process commonly referred to as the metastatic cascade. This cascade encompasses local invasion, intravasation into blood and/or lymphatic vessels, survival as circulating tumor cells (CTCs), extravasation, micrometastatic outgrowth, and ultimately macrometastatic colonization (2, 4–9). Each stage is shaped by context-dependent genetic alterations, reversible epigenetic programs, and reciprocal interactions with the tumor microenvironment. Importantly, epigenetic regulation operates not only during epithelial–mesenchymal transition (EMT) but across all stages of the metastatic cascade, coordinating dynamic and context-dependent cellular transitions (10). Consistent with this view, recent updates to the Hallmarks of Cancer framework introduced “unlocking phenotypic plasticity” and “nonmutational epigenetic reprogramming” as enabling characteristics of cancer, highlighting the pivotal role of epigenetic regulation in tumor progression and metastatic competence (9).

EMT is not a binary process but rather a dynamic and reversible spectrum that includes intermediate or hybrid epithelial–mesenchymal states. These hybrid phenotypes retain both epithelial and mesenchymal features and are increasingly recognized as critical drivers of metastatic plasticity (11). Tumor invasion represents the initial step of metastatic dissemination and involves the degradation of the basement membrane (BM) followed by the infiltration of adjacent normal tissue. In carcinomas of epithelial origin—which account for more than 90% of human cancers—this process is closely associated with EMT (12, 13). During EMT, tumor cells undergo profound phenotypic reprogramming characterized by the loss of cell–cell adhesion, disruption of apico–basal polarity, and acquisition of migratory and invasive properties. These changes are accompanied by the downregulation of epithelial markers, most notably *E-cadherin*, and the upregulation of mesenchymal markers such as *N-cadherin* and *vimentin (*13, 14). The suppression of E-cadherin (CDH1) expression has been shown in functional experimental studies to be orchestrated by pleiotropic transcription factors including SNAIL, SLUG, TWIST, ZEB1, and ZEB2, which act through transcriptional and post-transcriptional mechanisms, often in cooperation with epigenetic modifiers (13, 14). Concurrently, activation of matrix metalloproteinases (MMPs) promotes BM disruption and extracellular matrix (ECM) remodeling, thereby facilitating tumor cell invasion. Beyond EMT, epigenetic mechanisms continue to regulate tumor cell survival during detachment and subsequent stages of metastatic progression (15).

Following detachment from the primary tumor and survival in circulation, tumor cells undergo additional epigenetically regulated processes that facilitate extravasation and colonization (16). To successfully metastasize, tumor cells must acquire resistance to anoikis through extensive biochemical and transcriptional reprogramming (17). Anoikis-resistant cells can then intravasate into the vasculature, circulate as CTCs, and evade immune surveillance. However, only a minute fraction of these circulating cells survives and ultimately extravasates into distant tissues. At secondary sites, disseminated tumor cells frequently undergo mesenchymal–epithelial transition (MET), a reversal of EMT-associated features, which facilitates cellular adaptation, proliferation, and the formation of micrometastases that may progress to overt macrometastatic lesions (18, 19). MET represents the reverse process of EMT, enabling mesenchymal cells to reacquire epithelial characteristics during metastatic colonization (20).

Collectively, metastasis should not be viewed as an EMT-centered event, but rather as a continuous and epigenetically regulated process spanning multiple interconnected stages (21). In this framework, EMT, MET, anoikis resistance, and metastatic colonization constitute interconnected and context-dependent cellular states governed by nonmutational epigenetic reprogramming, which enables phenotypic plasticity and adaptive fitness during metastatic progression. Accordingly, epigenetic regulation emerges as a central determinant of metastatic initiation, dissemination, survival, and outgrowth.

In this review, we provide a comprehensive synthesis of the epigenetic mechanisms that regulate metastatic progression, with a particular focus on EMT-associated transcriptional programs, chromatin-based regulatory networks, and non-coding RNA circuits. Where appropriate, we distinguish between correlative associations and experimentally validated causal mechanisms to clarify the strength of evidence. We further discuss the translational implications of metastasis-associated epigenetic alterations as clinically relevant biomarkers and therapeutic targets, emphasizing their potential integration into precision oncology strategies.

## Molecular basis of epigenetic regulation

Epigenetic mechanisms enable dynamic and heritable changes in gene expression without alterations in the underlying DNA sequence, thereby allowing cells to adapt transcriptional programs in response to developmental cues and environmental stimuli (22). These processes can lead to the activation, repression, or complete silencing of gene expression and are mediated through interconnected regulatory pathways, including DNA methylation, histone modifications, chromatin remodeling, noncoding RNAs, and higher-order chromosomal organization (23). Due to their reversible and context-dependent nature, epigenetic mechanisms provide a flexible regulatory framework that governs cellular identity, function, and plasticity.

Epigenetic regulation plays a fundamental role in cellular development, differentiation, and homeostasis, and its dysregulation is a hallmark of numerous diseases, particularly cancer (23, 24). DNA methylation involves the transfer of a methyl group from S-adenosyl methionine to the 5-carbon position of cytosine residues within CpG dinucleotides, a reaction catalyzed by DNA methyltransferases (DNMTs) in CpG-rich genomic regions (25). In cancer cells, aberrant hypermethylation of gene promoter regions suppresses transcription by inhibiting transcription factor binding or facilitating the recruitment of transcriptional repressors, contributing to the functional inactivation of tumor suppressor genes (26, 27). Conversely, global or regional DNA hypomethylation, particularly within regulatory elements or repetitive sequences, promotes genomic instability and may lead to oncogene reactivation, thereby facilitating tumor initiation and progression (28, 29).

In addition to DNA methylation, chromatin structure and transcriptional activity are extensively regulated by post-translational modifications (PTMs) of histone proteins. Chromatin exists in transcriptionally active euchromatin and transcriptionally repressed heterochromatin states, and transitions between these states are governed by covalent histone modifications (30–32). These modifications, including methylation, acetylation, phosphorylation, ubiquitination, and SUMOylation, influence gene expression either by altering chromatin compaction or by serving as docking platforms for regulatory protein complexes (31–33). Histone modifications are dynamically regulated by specific enzymes, such as histone methyltransferases and demethylases, as well as histone acetyltransferases and deacetylases (30–32). In the context of cancer, dysregulated histone modification patterns contribute to altered transcriptional programs that promote tumor initiation, invasion, and metastatic progression.

Beyond covalent histone modifications, ATP-dependent chromatin remodeling complexes play a critical role in regulating DNA accessibility and transcriptional control. Among these, the SWI/SNF (SWItch/Sucrose Non-Fermentable) complex modulates nucleosome positioning to regulate transcription, maintain genomic stability, and facilitate DNA repair (34–36). Recent studies also highlight cooperation between SWI/SNF complexes and lncRNAs in modulating EMT-related promoters, reinforcing transcriptional programs that drive metastasis in various cancers (37). Genetic alterations affecting SWI/SNF complex components have been identified in multiple cancer types, underscoring the tumor suppressor functions of this complex and its involvement in oncogenic transcriptional reprogramming (38).

Noncoding RNAs represent another major layer of epigenetic regulation. RNA interference is a post-transcriptional gene silencing mechanism mediated by small RNA molecules that target complementary mRNA transcripts for degradation or translational repression (39, 40). MicroRNAs (miRNAs), the most extensively studied class of small noncoding RNAs, regulate gene expression through sequence-specific interactions and are involved in key physiological and pathological processes, including cell fate determination, differentiation, and tumor progression (41). In cancer, dysregulated miRNA expression contributes to aberrant gene regulation by enhancing the suppression of tumor suppressor genes or relieving repression of oncogenes (42, 43). Consequently, miRNAs play critical regulatory roles throughout tumor initiation, invasion, and the metastatic cascade and are actively being explored as diagnostic biomarkers and therapeutic targets (41).

Long noncoding RNAs (lncRNAs), although lacking protein-coding capacity, regulate gene expression at epigenetic, transcriptional, and post-transcriptional levels and have emerged as key modulators of carcinogenesis and metastatic behavior (44). Depending on their molecular context and interacting partners, lncRNAs may function either as promoters or suppressors of tumor progression and metastasis.

Circular RNAs (circRNAs) are covalently closed, single-stranded RNA molecules that exhibit high stability and regulatory potential (45). Although their functions are still being elucidated, circRNAs have been shown to act as miRNA sponges, interact with RNA-binding proteins, and modulate transcriptional and translational processes. Aberrant circRNA expression patterns have been increasingly associated with cancer development and metastatic progression, highlighting their emerging roles as epigenetic regulators and potential clinical biomarkers (45, 46).

Importantly, epigenetic mechanisms do not function as independent regulatory layers but rather operate in a highly coordinated and interconnected manner. DNA methylation, histone modifications, and non-coding RNAs frequently cooperate to establish and maintain transcriptional states. For example, DNA methylation can recruit methyl-CpG-binding proteins that interact with histone deacetylases and histone methyltransferases to reinforce chromatin compaction, while non-coding RNAs can guide chromatin-modifying complexes to specific genomic loci (47, 48). This multilayered crosstalk enables precise and dynamic regulation of gene expression programs underlying EMT and metastatic progression.

## Molecular regulation of EMT

Epithelial cells reside on the BM, exhibit apical–basal polarity, and are interconnected through specialized cell–cell junctions, including tight junctions, adherens junctions, and desmosomes, while being anchored to the BM via integrin-mediated hemidesmosomes (49). The structural integrity and characteristic morphology of epithelial tissues are maintained by coordinated interactions between surface adhesion molecules and the cytoskeletal network (50). These junctional complexes stabilize cell–cell and cell–matrix interactions, thereby restricting individual cell motility and preserving tissue architecture (50). Adherens junctions are linked to cortical actin filaments, desmosomes to intermediate filaments, and tight junctions localize to the apical–lateral membrane domain, where they play a crucial role in maintaining epithelial polarity (51). Disruption of epithelial junctional integrity—including adherens junctions, desmosomes, and gap junctions—leads to the loss of cell–cell adhesion, detachment from the BM, breakdown of contact inhibition, and dissolution of apical–basal polarity. These changes promote the emergence of cells with mesenchymal features and enhanced migratory capacity (52). Importantly, EMT is not a binary process; depending on the extent of phenotypic reprogramming, cells may acquire hybrid epithelial–mesenchymal states, allowing reversible transitions and dynamic plasticity between phenotypes (53).

During EMT, epithelial markers are progressively downregulated, while mesenchymal markers are upregulated. E-cadherin, a key component of adherens junctions, is widely regarded as a central molecular hallmark of EMT. Concomitantly, the expression of other epithelial markers, including cytokeratins (CK19, CK18, CK8), occludin, and desmoplakin, is reduced, whereas mesenchymal markers such as N-cadherin, vimentin, α-smooth muscle actin (α-SMA), fibronectin, and vitronectin are induced (54). Suppression of E-cadherin expression is primarily mediated at the transcriptional level by EMT-associated transcription factors (EMT-TFs). Members of the zinc-finger transcription factor family, including SNAIL (SNAIL and SLUG), TWIST1/2, and ZEB1/ZEB2 (SIP1), bind to E-box elements within the CDH1 promoter, leading to transcriptional repression, reduced cell–cell adhesion, and increased cellular plasticity (11, 54). In epithelial cells, β-catenin is normally sequestered at adherens junctions through its association with E-cadherin. Loss of E-cadherin disrupts this complex, allowing β-catenin to accumulate in the cytoplasm and subsequently translocate to the nucleus, where it functions as a transcriptional regulator of genes involved in pathways such as Wnt signalling (55, 56). In parallel, growth factor–activated pathways, particularly RAS signalling, can initiate EMT by modulating EMT-TF expression (57). RAS activation promotes the production of transforming growth factor-β (TGF-β), ECM components, MMP2, MMP9, and integrin receptors, while also stimulating the secretion of epidermal growth factors (EGF), platelet-derived growth factors (PDGF), and pro-inflammatory cytokines, all of which contribute to EMT induction (58). Within the canonical TGF-β/SMAD pathway, TGF-β–activated SMAD complexes directly bind to the promoter regions of SNAI1, TWIST1/2, and ZEB1, leading to increased transcription of these EMT-TFs and initiation of the EMT program (59). Accumulating evidence further indicates that NOTCH signalling has been shown to promote EMT in experimental models and metastatic progression by upregulating EMT-TFs such as SNAIL and SLUG, while simultaneously remodeling the tumor microenvironment (60, 61). Additional signalling pathways, including hypoxia-inducible factor 1α (HIF1α) and tumor necrosis factor-α (TNF-α), also regulate EMT during tumor progression by inducing the expression of SNAIL, SLUG, and TWIST (62, 63). HIF1α may further potentiate EMT through crosstalk with TGF-β signalling. Collectively, the integration of these pathways results in loss of epithelial junctional integrity, cytoskeletal reorganization, and enhanced proteolytic activity, ultimately driving invasive behavior, anoikis resistance, and therapy-resistant tumor phenotypes (62). Key pathways are summarized in **Table 1**.

**Table 1:** Key molecular pathways regulating EMT and their functional consequences.

Molecule/Pathway	Mechanism/Target	Outcome/Effect	References
E-cadherin	EMT-TFs (SNAIL, SLUG, TWIST, ZEB1/2) repress by binding to the promoter region	Loss of cell-cell adhesion, initiation of EMT	(11, 54)
β-catenin	Loss of E-cadherin → dissociates from complex → migrates to nucleus → activates Wnt target genes	Cell proliferation contributes to EMT	(55, 56)
CK8, CK18, CK19	Expression decreases	Loss of epithelial features	(54)
Occludin, Desmoplakin	Expression decreases	Disruption of tight junctions and epithelial integrity	(51, 54)
N-cadherin, Vimentin	Expression increases	Transition to mesenchymal phenotype, cell motility	(54)
α-SMA	Expression increases	Cell contractility increases, invasion ability	(54)
Fibronectin, Vitronectin	Increased synthesis	Cell-ECM interactions and increased migration	(54)
SNAIL, SLUG	Represses CDH1 gene	Loss of epithelial markers, initiation of EMT	(11, 54)
TWIST1/2	Represses epithelial genes and activates mesenchymal genes	Cellular plasticity, EMT progression	(54)
ZEB1/ZEB2 (SIP1)	Binds to CDH1 gene and suppresses expression	Loss of cell polarity	(11, 54)
TGF-β/SMAD	SMAD2/3 → complex with SMAD4 → binding to SNAIL, ZEB, TWIST promoter	Activation of EMT-TFs, EMT initiation	(59)
Wnt/β- catenin	β-catenin passes into the nucleus → activates target genes	Cell proliferation, invasion	(55, 56)
RAS/MAPK	Activated by growth factors (EGF, PDGF) → EMT-TF expression increases	Change in the cytoskeleton, increased movement	(57, 58)
NOTCH	Increase SLUG and SNAIL expression	Tumor microenvironment is reshaped; metastasis increases	(60, 61)
HIF-1α	Induces EMT-TFs under hypoxic conditions; interacts with TGF-β	Cell invasion, drug resistance, anoikis resistance	(62, 63)
TNF-α	Increase factors like SNAIL, SLUG, TWIST	Inflammation-associated EMT activation	(62)
MMP2, MMP9	Degrades ECM components	Facilitates cell invasion into surrounding tissues	(58)
Integrins	Reorganization of ECM and cell junctions	Cell migration and transition to mesenchymal phenotype	(49, 58)

→: Causal or regulatory relationship.

## Epigenetic regulation in EMT

### Part I. DNA methylation–based regulation of EMT and metastasis

Aberrant DNA methylation is a key epigenetic mechanism associated with tumor initiation, progression, and metastasis by promoting chromosomal instability, silencing tumor suppressor and DNA repair genes through promoter hypermethylation, and inducing oncogene activation via global or regional hypomethylation (15, 64). These methylation alterations reshape transcriptional programs that facilitate phenotypic plasticity, invasion, and metastatic competence.

DNA methylation is catalyzed by the DNMT family, which comprises five members: DNMT1, DNMT2, DNMT3A, DNMT3B, and DNMT3L (65). Among these, DNMT1, DNMT3A, and DNMT3B are the principal enzymes responsible for CpG island methylation. DNMT1 preferentially recognizes hemimethylated DNA following replication, thereby maintaining established methylation patterns, whereas DNMT3A and DNMT3B mediate both maintenance and *de novo* DNA methylation, particularly during development and disease states (15, 65, 66). Increasing evidence implicates DNMT dysregulation in EMT-associated transcriptional repression. For instance, elevated DNMT3B expression has been shown in experimental studies to induce hypermethylation of the CDH1 promoter, leading to E-cadherin silencing and EMT promotion (67). Similarly, DNMT3B7, an aberrantly expressed DNMT isoform detected in multiple cancer types, suppresses CDH1 expression in breast cancer cells via promoter hypermethylation while enhancing invasiveness and metastatic potential through increased β-catenin activity (68, 69). Functional evidence further supports the causal role of DNA methylation in EMT regulation. In breast cancer cell lines, aberrant DNMT3B activation induces CpG island hypermethylation within the CDH1 promoter, whereas treatment with the demethylating agent 5-azacytidine restores gene expression, directly linking promoter methylation to E-cadherin repression (70). Moreover, TGF-β, a potent EMT inducer, has been shown to upregulate both the expression and enzymatic activity of DNMT1, DNMT3A, and DNMT3B in ovarian cancer cells. This coordinated DNMT activation results in methylation-dependent repression of EMT-related genes such as CDH1 and COL1A1, while pharmacological DNMT inhibition reverses these methylation patterns and suppresses EMT (71). DNA methylation does not act in isolation but rather cooperates with other epigenetic mechanisms, including histone modifications and noncoding RNA–mediated regulation. In this context, global DNA hypomethylation has emerged as a driver of tumor progression comparable in impact to gene-specific promoter hypermethylation, particularly during early stages of lung carcinogenesis (72). Supporting this notion, treatment with the DNMT inhibitor decitabine induces upregulation of the miR-200 family and downregulation of ZEB1/2 in lung cancer cell lines, leading to the reversal of TGF-β–induced EMT (73). These findings highlight the functional interplay between DNA methylation and miRNA-mediated control of EMT-associated transcriptional networks. Methyl-CpG-binding domain (MBD) proteins provide a mechanistic link between DNA methylation and chromatin-based gene repression by recognizing methylated CpG sites and recruiting histone deacetylases and histone methyltransferases to enforce transcriptional silencing (74). Elevated MBD2 expression has been reported in renal cell carcinoma (RCC) tissues and cell lines and is associated with increased expression of mesenchymal markers N-cadherin and vimentin, concomitant loss of E-cadherin, and induction of EMT. Clinically, MBD2 overexpression correlates with poor prognosis, reduced survival, and enhanced migratory, invasive, and metastatic potential in RCC (75, 76). DNA methylation–dependent regulation of noncoding RNAs further contributes to EMT and metastasis. In cervical cancer, the tumor-suppressive miRNA miR-138 is highly expressed in normal tissues but becomes epigenetically silenced through promoter hypermethylation in cancer cells, resulting in EMT induction and increased metastatic capacity. Restoration of miR-138 expression by decitabine treatment or agomiR-138 transfection inhibits EMT and significantly reduces cell proliferation, invasion, and metastasis (77). Collectively, these findings demonstrate that aberrant DNA methylation patterns—encompassing both promoter-specific hypermethylation and global hypomethylation—play a central role in EMT initiation and metastatic progression across diverse cancer types. Notably, while several observations are based on clinical correlations, others are supported by functional experimental studies, underscoring the importance of distinguishing between association and causality in epigenetic regulation of metastasis (78). Representative examples of DNA methylation–based epigenetic regulation of EMT and metastasis are summarized in **Table 2A**.

**Table 2A:** DNA methylation–based epigenetic regulation of EMT and metastasis.

Gene/Tf/Molecule	Epigenetic mechanism	Modification type	Enzyme/Complex/Pathway	Type of cancer	References
E-Cadherin	DNA hypermethylation	Promoter hypermethylation	DNMT3B, DNMT3B7	HNSCC, Breast cancer	(67, 68, 70)
E-Cadherin, COL1A1	DNA methylation	Promoter hypermethylation by TGF-β effect	DNMT1, DNMT3A, DNMT3B	Ovarian cancer	(71)
Global DNA	DNA hypomethylation	Global hypomethylation + gene specific hypermethylation	DNMTs	Lung cancer	(72)
ZEB1/ZEB2	DNA methylation (miRNA mediated)	miR-200 repression → ZEB1 ↑	–	Lung cancer	(73)
MBD2	DNA methylation + MBD protein	Binding to methylated DNA and EMT induction	MBD2 + HDACs, HMTs	Renal cell carcinoma	(75, 76)
miR-138/ZEB2	DNA methylation	miR-138 promoter methylation	DNMTs	Cervical cancer	(77)

→: Causal or regulatory relationship; ↑: Increased expression or activation; ↓: Decreased expression or inhibition.

### Part II. Regulation of EMT through histone modifications

Similar to gene-specific DNA methylation, post-translational covalent modifications of histone proteins—most notably methylation and acetylation—play a central role in the epigenetic regulation of EMT by dynamically reorganizing chromatin structure (79). Depending on their type and genomic context, histone PTMs can either activate or repress gene transcription. In general, histone acetylation and demethylation are associated with transcriptional activation, whereas deacetylation and methylation are linked to gene repression (80). Histone acetylation and deacetylation are catalyzed by lysine acetyltransferases (KATs/HATs) and lysine deacetylases (KDACs/HDACs), respectively, while histone methylation and demethylation are mediated by histone methyltransferases (HMTs) and histone demethylases (HDMs) targeting lysine and arginine residues (81). In breast cancer cell lines, SNAI1 was shown to interact with the histone demethylase LSD1, thereby maintaining transcriptional repression of E-cadherin (82). Through demethylation of the activating H3K4me2 mark, LSD1 suppresses active chromatin states and promotes EMT (83). Similarly, in lung and pancreatic cancer models, upregulation of the lysine demethylase KDM2B resulted in reduced expression of epithelial markers, including CDH1 and miR-200, particularly in the presence of TGF-β signaling (84). EZH2, the catalytic subunit of the Polycomb repressive complex 2 (PRC2), mediates transcriptional silencing through deposition of the repressive H3K27me3 mark (15). In ovarian cancer cells, EZH2 overexpression suppresses TIMP2 expression via coordinated H3K27me3 deposition and DNA methylation, thereby enhancing invasion and migratory capacity (85). Another key regulator, SETD1A, catalyzes mono-, di-, and tri-methylation of H3K4 and functions as a transcriptional activator (86, 87). In gastric cancer cells, SETD1A overexpression increased SNAIL expression by elevating H3K4 methylation at its promoter, inducing EMT. Conversely, SETD1A knockdown restored epithelial characteristics by upregulating E-cadherin and downregulating mesenchymal markers such as N-cadherin, Vimentin, Fibronectin, and α-SMA (88). In non-small cell lung cancer, SETD1A was further shown to interact with β-catenin, activating the Wnt/β-catenin pathway and promoting transcription of NEAT1 and EZH2 in an H3K4me3-dependent manner (86). Aberrant activation of Wnt/β-catenin signaling is a well-established driver of EMT, tumor progression, and metastasis (89). Consistently, SETD1A-mediated H3K4me3 enrichment at transcription start sites of multiple oncogenes promoted cell proliferation and EMT via Wnt and TGF-β signaling in lung cancer models. In SETD1A-silenced xenografts, reduced expression of EMT-related genes (CDH2, VIM, ZEB1, SNAIL) and increased CDH1 expression were observed, alongside decreased levels of epigenetic regulators (DNMT1, SETDB1, HDAC1) and oncogenic factors (MYC, GLI1, FOXM1, STAT3, FAK) (90). These findings underscore the global role of SETD1A-driven H3K4me3 in coordinating oncogenic and EMT-associated transcriptional programs. Histone-modifying enzymes also regulate EMT transcription factors in metastatic dissemination. In lung adenocarcinoma brain metastases, EMT-TFs including SLUG, TWIST, ZEB1, and FOXC2 were shown to be differentially regulated by MLL4, UTX, and EZH2. Specifically, MLL4 suppressed SLUG, UTX inhibited ZEB1, and EZH2 repressed TWIST, collectively influencing EMT, migration, and metastatic potential (91).

In hepatocellular carcinoma (HCC), overexpression of SNAIL2 has been frequently reported (92). During TGF-β-induced EMT, SNAIL2 recruits G9a and HDACs to the E-cadherin promoter, increasing H3K9 methylation and promoting deacetylation of H3K56 and H3K4, thereby facilitating EMT, cell migration, and metastasis (93). Comparable mechanisms have been described in lung cancer cells undergoing TGF-β-induced EMT (94). In gastric cancer, the long non-coding RNA HOTAIR directs PRC2 to the E-cadherin promoter, inducing H3K27me3-mediated repression while preventing CBP-mediated H3K27 acetylation, ultimately promoting EMT (95). Recent studies indicate that lncRNAs and chromatin remodelers such as SWI/SNF complexes cooperate to modulate chromatin accessibility at EMT-related promoters, reinforcing transcriptional programs that drive metastasis in multiple cancer types (Zhuang et al., 2024; Li et al., 2024). Such chromatin-modifying events often operate in concert with ATP-dependent remodelers to fine-tune nucleosome positioning and enhancer–promoter interactions, highlighting the integration of multiple epigenetic layers in EMT regulation (96). DOT1L, the sole methyltransferase responsible for H3K79 methylation, is associated with transcriptional activation (97). In breast cancer, DOT1L interacts with the c-Myc–p300 complex to activate EMT-TFs (SNAIL, ZEB1, ZEB2) via coordinated H3K79 methylation and histone acetylation, thereby inducing EMT and cancer stem cell–like properties (98). Crosstalk between epigenetic regulation and signaling pathways is further exemplified by PI3K/AKT and Wnt/β-catenin signaling in gastric cancer, where inhibition of these pathways reduced H3K4 and H3K27 acetylation and suppressed TWIST expression, highlighting their role in EMT maintenance (99). Beyond histone modifications, phosphorylation serves as a critical regulatory PTM influencing EMT-related signaling cascades and transcription factor stability (100, 101). In prostate cancer cells, protein kinase D1 (PDK1) phosphorylates SNAIL at Ser11, disrupting recruitment of the Sin3A–HDAC1/2 complex and reversing EMT, positioning PDK1 as a tumor suppressor (102). GSK3-β also plays a dual role in EMT regulation: its phosphorylation and inactivation by PI3K/AKT signaling stabilizes β-catenin and SNAIL, promoting EMT, while active GSK3-β facilitates their degradation (103, 104). This mechanism has been implicated in HIF-1α-mediated EMT induction in oral squamous cell carcinoma (104) and ERK/MAPK-driven EMT via Fas signaling (105). Additionally, phosphorylation of TWIST1 at Thr125 and Ser127 is essential for its dimerization and pro-metastatic activity in prostate cancer (106).

The ubiquitin–proteasome system constitutes another critical layer of EMT regulation by controlling protein stability and turnover (107–109). During TGF-β-induced EMT, ERK1/2-mediated phosphorylation of the E3 ligase Smurf1 enhances RhoA ubiquitination and degradation, promoting cytoskeletal remodeling and EMT progression (110). In HCC, DCAF15 functions as a tumor suppressor by promoting ubiquitin-dependent degradation of ZEB1 (109). The balance between ubiquitination and deubiquitination is further exemplified by the opposing actions of USP39 and TRIM26 in regulating ZEB1 stability in HCC (111). In gastric cancer, PLAGL2 (pleomorphic adenoma gene-like 2)-driven upregulation of USP37 stabilizes phosphorylated SNAIL1, thereby promoting EMT, invasion, and metastasis (112). Similarly, wild-type p53 suppresses EMT in ovarian cancer by facilitating Pirh2-mediated ubiquitination and degradation of TWIST1 (113).

SUMOylation, another reversible PTM involving small ubiquitin-like modifiers, also contributes to EMT regulation (114, 115). In metastatic prostate cancer, SENP1 depletion increased E-cadherin and SMAD4 expression and attenuated TGF-β-induced EMT (116). In hepatoma cells, hepatitis B virus X protein-induced IGF-II promoted EMT through SUMOylation-dependent loss of E-cadherin and upregulation of TWIST (117). In gastric cancer, the redox-sensitive SUMO protease SENP3 enhances EMT by de-SUMOylating FOXC2 under oxidative stress, thereby increasing its transcriptional activity toward mesenchymal genes (118). Collectively, these findings highlight that, beyond DNA methylation, histone PTMs and related regulatory mechanisms—including phosphorylation, ubiquitination, and SUMOylation—play indispensable roles in shaping chromatin architecture and transcriptional programs that govern EMT and metastatic progression. Key histone modifications and associated post-translational regulatory events involved in EMT are summarized in **Table 2B**.

**Table 2B:** Histone modifications and post-translational regulation of EMT.

Gene/Tf/Molecule	Epigenetic mechanism	Modification type	Enzyme/Complex/Pathway	Type of cancer	References
SNAI1	Histone demethylation	H3K4me2 demethylation	LSD1 (KDM1A)	Breast cancer	(82, 83)
E-Cadherin, mir-200	Histone demethylation	H3 demethylation	KDM2B	Lung, pancreas cancer	(84)
TIMP2	Histone + DNA methylation	H3K27me3 + DNA methylation	EZH2 (PRC2 complex)	Ovarian cancer	(15, 85)
SNAIL	Histone methylation	H3K4me1/2/3	SETD1A	Gastric cancer	(88)
NEAT1, EZH2, SNAIL	Histone methylation	H3K4me3-dependent	SETD1A + β-catenin	Non-small cell lung carcinoma	(86, 90)
SLUG, ZEB1, TWIST, FOXC2	Histone methylation	H3K4me3, H3K36me3, H3K27me3	MLL4, UTX, EZH2	Brain metastasis	(91)
E-Cadherin	Histone methylation, deacethylation	H3K9me2 + H3K4/H3K56 deacetylation	G9a, HDAC1/2/3 + SNAIL2	Hepatocellular carcinoma, Lung cancer	(92, 93)
E-Cadherin	Histone methylation	H3K27me3	HOTAIR via PRC2	Gastric cancer	(95)
DOT1L (via SNAIL, ZEB1/2)	Histone methylation + acetylation	H3K79me, H3ac	DOT1L + c-Myc-p300 complex	Breast cancer	(97, 98)
TWIST	Histone acetylation	H3K4ac, H3K27ac	PI3K/AKT & Wnt/β-catenin pathways	Gastric cancer	(99)
SNAIL	Phosphorylation	Ser11 phosphorylation	PDK1	Prostate cancer	(102)
β-Catenin	Phosphorylation	↓ GSK3-β-mediated degradation	PI3K/AKT → GSK3-β	Oral squamous cell carcinoma, Prostate	(103–105)
TWIST1	Phosphorylation	Thr125, Ser127	Unknown kinase	Prostate cancer	(106)
ZEB1	Ubiquitination	Degradation	DCAF15 E3 ligase	Hepatocellular carcinoma	(109)
SMURF1	Ubiquitination	Thr223 phosphorylation, RhoA degradation	ERK1/2	Various cancers	(110)
ZEB1	Ubiquitination/ De ubiquitination	Stabilization or degradation	USP39, TRIM26	Hepatocellular carcinoma	(111)
SNAIL1	De ubiquitination	GSK3β-dependent	USP37 (via PLAGL2)	Gastric cancer	(112)
TWIST1	Ubiquitination	Proteasomal degradation	p53 + Pirh2 (E3 ligase)	Ovarian cancer	(113)
E-cadherin, SMAD4	SUMOylation	↓ SUMOylation	SENP1	Prostate cancer	(116)
E-cadherin	SUMOylation	↑ SUMOylation (via HBx)	IGF-II, HBx-induced SUMO	Hepatoma cells	(117)
FOXC2	SUMOylation	↓ SUMOylation	SENP3	Gastric cancer	(118)

→: Causal or regulatory relationship; ↑: Increased expression or activation; ↓: Decreased expression or inhibition.

### Part III: miRNA-mediated control of EMT and metastasis

MicroRNAs (miRNAs) constitute a major class of epigenetic regulators that have been reported to play critical roles in carcinogenesis and cancer progression by modulating EMT, oncogenic signaling pathways, and metastatic dissemination (119). miRNAs are small, endogenous non-coding RNAs that post-transcriptionally suppress gene expression through direct binding to complementary sequences within the 3′ untranslated regions (3′UTRs) of target mRNAs (120). Through this mechanism, miRNAs fine-tune the expression of EMT-associated transcription factors, signaling components, and structural proteins, thereby influencing cellular plasticity and metastatic potential.

A prototypical example of miRNA-mediated EMT regulation, supported by functional experimental studies, is the reciprocal feedback loop between ZEB1 and the miR-200 family (121). In colorectal cancer (CRC), ZEB1 directly binds to the promoter regions of miR-200 family members (miR-200a, miR-200b, miR-200c, miR-141, and miR-429), transcriptionally repressing their expression. Conversely, elevated miR-200 levels post-transcriptionally suppress ZEB1 by targeting its 3′UTR. Depending on upstream cues, this bistable regulatory circuit can drive cells toward either an epithelial or mesenchymal phenotype, highlighting its central role in EMT plasticity (122). In non-small cell lung carcinoma (NSCLC), miR-128-3p has been shown to disrupt EMT regulation through an indirect mechanism involving miRNA biogenesis. By inhibiting the core processing enzymes Drosha and Dicer, miR-128-3p reduces the global abundance of EMT-suppressive miRNAs, resulting in increased expression of EMT transcription factors such as SNAIL and ZEB1 and facilitating EMT progression (123). Similarly, miR-142 functions as a tumor suppressor by directly targeting TGF-β signaling and attenuating EMT under physiological conditions (124). In HCC, however, hypermethylation of the miR-142 promoter leads to its silencing, thereby enhancing TGF-β signaling and promoting EMT and tumor progression (125). Several miRNAs have also been implicated in EMT regulation across diverse cancer types through direct modulation of epithelial and mesenchymal markers. In tongue squamous cell carcinoma, miR-137 expression is markedly reduced compared with normal tissue. Restoration of miR-137 expression has been shown in experimental studies to increase E-cadherin levels while suppressing N-cadherin, Vimentin, and SNAIL, effectively inhibiting EMT (126). In HCC, downregulation of the histone demethylase KDM4B induces hypermethylation of the miR-615-5p promoter, resulting in reduced miR-615-5p expression. This epigenetic silencing leads to upregulation of the Ras-related protein RAB24, thereby promoting EMT, invasion, and metastasis (127). Epigenetic regulation of miRNAs themselves further reinforces EMT-associated transcriptional programs. In invasive breast cancer cell lines, ectopic expression of TWIST induces DNA methylation of the miR-200c and miR-141 promoters, resulting in their transcriptional repression and subsequent EMT induction *in vitro*. These findings establish DNA methylation as a key regulatory mechanism governing miR-200 family expression during EMT (128). In metastatic breast cancer cells, miR-203 has emerged as a context-dependent regulator of EMT. During TGF-β-induced EMT, SNAI2 binds to the miR-203 promoter and suppresses its expression. Concurrently, alterations in miR-200 family expression modulate downstream EMT targets. Notably, restoration of miR-203 indirectly influences ZEB1 and ZEB2 expression through modulation of the miR-200 family, underscoring the complexity of miRNA-mediated regulatory networks in EMT and metastasis (129).

Similarly, in CRC tissue samples that subsequently develop liver metastasis, the expression levels of miR-200c and miR-141 are significantly lower compared with those observed in metastatic liver lesions. Recent studies consistently demonstrate that the re-expression of these miRNAs in metastatic tissues is associated with hypomethylation of their promoter regions. While reduced miR-200c expression in primary CRC tissues promotes EMT through derepression of its target genes *ZEB1* and *ZEB2*, metastatic liver tissues exhibit downregulation of these EMT drivers, accompanied by decreased Vimentin expression and restored E-cadherin levels. This shift reflects a MET, highlighting the dynamic and reversible nature of EMT during metastatic colonization (130). In NSCLC, reduced miR-193a expression has been implicated in metastatic progression by promoting cell proliferation, facilitating TGF-β-induced EMT, and modulating the WT1–E-cadherin signaling axis (131). In pancreatic ductal adenocarcinoma, multiple miRNAs—including miR-34a, miR-141, miR-148a, miR-200b, miR-200c, and miR-655—have been identified as direct regulators of *ZEB1*. These miRNAs function as negative regulators of EMT and are frequently dysregulated during tumor progression (132). More recently, miR-4287 has emerged as a novel EMT-associated miRNA in prostate cancer. miR-4287 is overexpressed in prostate cancer cells and has been shown to suppress EMT by increasing E-cadherin expression while reducing Vimentin levels. Accumulating evidence further indicates that miR-4287 downregulates CD44, a key cancer stem cell marker, thereby limiting stemness-associated traits. Importantly, deletion of chromosome region 8p, which harbors the tumor suppressor gene *NKX3.1*, results in the loss of several miRNAs, including miR-4287. Loss of miR-4287 has been associated with increased invasiveness and metastatic potential in prostate cancer cells, supporting its role as a tumor suppressor miRNA (133). Collectively, these studies highlight miRNAs as dynamic epigenetic modulators that integrate transcriptional, post-transcriptional, and epigenetic signals to fine-tune EMT programs during cancer progression. Key miRNA-mediated regulatory mechanisms involved in EMT and metastatic dissemination are summarized in **Table 2C**.

**Table 2C:** miRNA-mediated epigenetic control of EMT and metastasis.

Gene/Tf/Molecule	Epigenetic mechanism	Modification type	Enzyme/Complex/Pathway	Type of cancer	References
miR-200 family (200a/b/c, 141, 429)	miRNA regulation/DNA methylation	ZEB1 inhibition/CDH1 regulation/Promoter methylation	ZEB1/DNMTs	Colorectal, Breast (liver metastasis)	(122, 128, 130)
miR-128-3p	Inhibition of miRNA biogenesis	–	Targets Drosha and Dicer → ↓ EMT-suppressor miRNAs	Non-small cell lung carcinoma	(123)
miR-142	DNA methylation of miRNA	Promoter methylation	TGF-β, DNMTs	Hepatocellular carcinoma	(124, 125)
miR-137	Downregulation	–	E-Cad ↑, N-Cad/VIM/SNAIL ↓	Tongue squamous cell carcinoma	(126)
miR-615-5p	DNA methylation of miRNA	DNA methylation	RAB24 derepression	Hepatocellular carcinoma	(127)
miR-203	miRNA regulation/DNA methylation	–	SNAI2 ↓, ZEB1/2 ↑ (via miR-200 suppression)	Metastatic breast cancer	(129)
miR-193a	miRNA regulation	-	WT1-E-cadherin axis deregulation	Non-small cell lung carcinoma	(131)
miR-34a, -141, -148a, -200b/c, -655	Post-transcriptional repression	–	Target ZEB1 → EMT inhibition	Pancreatic ductal adenocarcinoma	(132)
miR-4287	Genomic deletion (Chr 8p)	-	CD44, Vimentin ↓; E-cadherin ↑	Prostate cancer	(133)

→: Causal or regulatory relationship; ↑: Increased expression or activation; ↓: Decreased expression or inhibition.

### Part IV: lncRNAs in epigenetic modulation and cancer metastasis

Advances in high-throughput sequencing technologies and transcriptome-wide analyses have revealed that, although the human genome is extensively transcribed, only approximately 2% of transcripts encode proteins (134, 135). In addition to established lncRNAs such as HOTAIR and MALAT1, very recent evidence highlights that lncRNAs modulate the cancer epigenome by recruiting chromatin modifiers and shaping chromatin accessibility, thereby driving metastasis and therapeutic resistance (136). lncRNAs are defined as RNA transcripts longer than 200 nucleotides with limited or no protein-coding potential (137). ncRNAs are involved in a wide range of physiological and pathological processes, including cell growth, differentiation, development, and carcinogenesis. In cancer, lncRNAs can function either as oncogenic drivers or tumor suppressors; however, dysregulated lncRNA expression has been reported to influence not only tumor proliferation and metastasis but also EMT, drug resistance, and cancer stem cell phenotypes (138–140). MALAT1 is one of the most extensively studied lncRNAs and has been reported to be upregulated in patients with cutaneous T-cell lymphoma (CTCL). MALAT1 promotes EMT by inducing loss of E-cadherin and gain of N-cadherin expression. Silencing of MALAT1 results in reduced expression of stemness-associated transcription factors such as SOX2 and NANOG, while simultaneously increasing the levels of the tumor suppressor miR-124. Collectively, these findings indicate that MALAT1 overexpression is associated with EMT induction, acquisition of stem cell–like properties, and poor prognosis in CTCL (141). In CRC, aberrant lncRNA expression plays a critical role in tumorigenesis and metastatic progression. Upregulation of LINC00586, also known as BRAF-activated non-coding RNA (BANCR), leads to repression of ASXL1, a transcriptional regulator. Silencing of ASXL1 through LSD1-mediated H3K4me2 demethylation results in decreased E-cadherin expression and increased N-cadherin and Vimentin levels, thereby promoting EMT, invasion, and metastasis (142). Similarly, in bladder cancer, LINC02470 has been shown to regulate tumor progression through a competing endogenous RNA (ceRNA) mechanism. Both LINC02470 and SMAD3 are targeted by miR-143-3p; however, LINC02470 functions as a molecular sponge, protecting SMAD3 mRNA from miRNA-mediated degradation. As a result, enhanced SMAD3 expression facilitates TGF-β–induced activation of EMT transcription factors such as SNAIL and SLUG, thereby driving EMT and metastatic behavior (143). Epigenetic regulation of lncRNA expression itself also contributes to metastatic progression. In CRC cells, histone deacetylase 2 (HDAC2) suppresses the expression of lncRNA H19 by binding to SP1 and inducing deacetylation of H3K27 at the H19 promoter. In contrast, metastatic CRC tissues exhibit reduced HDAC2 expression accompanied by elevated H19 levels, which in turn enhance EMT and metastasis through upregulation of MMP14 (144). Recent studies further highlight that lncRNAs can recruit SWI/SNF complexes or other chromatin remodelers to specific genomic loci, thereby modulating chromatin accessibility and enhancer–promoter interactions to drive EMT and metastasis (37). The lncRNA MEG3 has been shown to interact with the chromatin regulator JARID2 (145). Under TGF-β stimulation, JARID2 recruits EZH2 to regulatory regions, leading to H3K27 trimethylation–mediated repression of E-cadherin, miR-200a, and miR-200c, while simultaneously increasing ZEB1 and ZEB2 expression (146). In lung cancer cells, overexpression of MEG3 has been reported to potentiate JARID2-dependent TGF-β–induced EMT and enhance metastatic potential (147). In HCC cells, elevated expression of the non-oncogenic lncRNA HCCL5 has been shown to promote EMT by upregulating EMT transcription factors including SNAIL, SLUG, ZEB1, and TWIST1, thereby facilitating TGF-β1–induced invasion and metastasis (148). These mechanisms often involve the recruitment of chromatin remodeling complexes, such as SWI/SNF or PRC2, to fine-tune transcriptional programs that govern metastatic plasticity, as reported in recent pan-cancer analyses (149). Conversely, lncRNA NKILA acts as a tumor suppressor in breast cancer. NF-κB–dependent TGF-β signaling induces NKILA expression, which in turn inhibits NF-κB activity, forming a negative feedback loop that suppresses EMT and metastatic dissemination (150, 151). Collectively, these findings highlight lncRNAs as critical epigenetic regulators that function as molecular scaffolds, guides, and decoys to coordinate chromatin modifiers and transcriptional programs governing EMT plasticity and metastatic progression. Representative lncRNAs involved in the epigenetic regulation of EMT and cancer metastasis are summarized in **Table 2D**.

**Table 2D:** lncRNAs in epigenetic modulation of EMT and metastasis.

Gene/Tf/Molecule	Epigenetic mechanism	Modification type	Enzyme/Complex/Pathway	Type of cancer	References
MALAT1	EMT regulation, miRNA interaction	-	E-cadherin ↓, N-cadherin ↑, miR-124 ↑	Cutaneous T-cell lymphoma	(141)
LINC00586/BANCR	Transcriptional repression	H3K4me2 demethylation	LSD1 → ASXL1 ↓, E-cadherin ↓, N-cadherin ↑	Colorectal cancer	(142)
LINC02470	ceRNA interaction	-	LINC02470/miR-143-3p/SMAD3 ceRNA axis → EMT-TFs ↑	Bladder cancer	(143)
H19	Promoter histone deacetylation	H3K27 deacetylation	HDAC2/SP1 → H19 ↑ → MMP14 ↑, EMT ↑	Colorectal cancer	(144)
MEG3	PRC2-mediated gene silencing	H3K27 methylation	JARID2 → EZH2 → miR-200a/200c ↓, ZEB1/2 ↑	Lung cancer	(145–147)
HCCL	TGF-β1-mediated EMT induction	–	SNAIL, SLUG, ZEB1, TWIST1 ↑	Hepatocellular carcinoma	(148)
NKILA	NF-κB feedback regulation, suppression	-	TGF-β → NKILA ↑ → NF-κB ↓ → EMT ↓	Breast cancer	(150, 151)

→: Causal or regulatory relationship; ↑: Increased expression or activation; ↓: Decreased expression or inhibition.

### Part V: Epigenetic regulation of molecules associated with anoikis resistance

Anoikis is a specialized form of programmed cell death that is triggered when cells lose appropriate contact with neighboring cells or the ECM, thereby serving as a critical barrier against inappropriate cell survival and metastatic dissemination. Under physiological conditions, anoikis contributes to tissue homeostasis by preventing displaced epithelial cells from colonizing ectopic sites. In contrast, resistance to anoikis—characterized by sustained activation of survival signaling pathways and suppression of apoptosis—is a defining feature of metastatic cancer cells and enables their survival during dissemination to distant organs (152, 153). Anoikis is initiated through the activation of caspase cascades downstream of two principal apoptotic pathways: (i) the intrinsic mitochondrial pathway and (ii) the extrinsic death receptor–mediated pathway (153, 154). The intrinsic pathway is regulated primarily by BH3-only proteins, whereas the extrinsic pathway is activated through binding of death ligands such as Fas ligand (FasL) or TRAIL to their corresponding receptors, leading to caspase-8 activation. Notably, caspase-8 also integrates signals from integrin-mediated adhesion pathways. Loss of ECM attachment disrupts integrin signaling, thereby triggering anoikis. Ultimately, both intrinsic and extrinsic pathways converge on the activation of executioner caspase-3, resulting in apoptotic cell death (154–156). Anoikis resistance arises from a combination of genetic alterations, epigenetic reprogramming, and aberrant microenvironmental signaling. These changes lead to constitutive activation of integrin- and growth factor–dependent survival pathways and inhibition of apoptotic mechanisms. Among these pathways, sustained activation of PI3K/AKT signaling—central to cell survival, metabolism, and stress adaptation—is considered a key driver of anchorage-independent growth and anoikis resistance (152). The neurotrophin receptor TrkB (tyrosine kinase B), activated by its ligand brain-derived neurotrophic factor (BDNF), promotes anoikis resistance through downstream activation of AKT, Src, and MAPK signaling pathways. In endometrial cancer, TrkB overexpression has been shown to upregulate TWIST expression, thereby linking EMT induction with enhanced anoikis resistance (157). Consistently, in ovarian cancer, TWIST1 overexpression—driven by active epigenetic marks such as H3K4me3 and H3K27ac—has been associated with increased expression of the anti-apoptotic protein BCL2. This FZD7–TWIST1–BCL2 axis has been implicated in anoikis resistance and poor clinical outcome (158). In a mouse model of gastric cancer, increased H3K4me3 enrichment was observed in TWIST1-positive cells, whereas TWIST1 degradation mediated by Suv39h1/h2-dependent H3K9me3 was associated with reduced survival signaling. These findings suggest that histone methylation dynamics at the TWIST1 locus may critically influence anoikis resistance and metastatic competence (159). Metabolic and stress-response pathways also contribute to anoikis resistance through epigenetic regulation. In MCF10A breast epithelial cells, reduced expression of TP53I11 (tumor protein p53–inducible protein 11) promotes anoikis resistance by inhibiting the AMPK–AKT/mTOR/p70S6K signaling axis in a TP53-independent manner (160). In gastric cancer, increased expression of the class III histone deacetylase SIRT1 is accompanied by downregulation of miR-204. Restoration of miR-204 expression reverses EMT and reduces anoikis resistance, whereas miR-204 suppression leads to SIRT1-mediated inactivation of LKB1, thereby enhancing EMT and anchorage-independent survival (161). MicroRNAs further fine-tune anoikis susceptibility in a context-dependent manner. miR-145-5p expression is reduced in primary CRC compared to metastatic CRC. Interestingly, overexpression of miR-145-5p increases anoikis sensitivity in primary tumors while paradoxically promoting anoikis resistance in metastatic CRC cells, highlighting stage-specific regulatory effects (162). In lung cancer, elevated TGF-β1 expression suppresses SH2B3 (Src homology 2–B3, also known as LNK). SH2B3 overexpression inhibits EMT and anoikis resistance by negatively regulating the JAK2/STAT3 and SHP2/Grb2/PI3K/AKT signaling pathways, whereas TGF-β1–mediated SH2B3 repression enhances migration, metastasis, and anchorage-independent survival (162). Collectively, resistance to anoikis and acquisition of anchorage-independent growth represent critical enabling features of metastatic progression. These processes are tightly controlled by epigenetic mechanisms that integrate chromatin modifications, non-coding RNA networks, and survival signaling pathways. Key epigenetic regulators implicated in anoikis resistance and metastatic survival are summarized in **Table 2E**.

**Table 2E:** Epigenetic regulation of anoikis resistance and anchorage-independent growth.

Gene/Tf/Molecule	Epigenetic mechanism	Modification type	Enzyme/Complex/Pathway	Type of cancer	References
TrkB	Transcriptional upregulation	-	BDNF → TrkB → AKT/Src/MAPK activation → TWIST ↑	Endometrial cancer	(157)
TWIST1	Histone modification	H3K4me3 ↑, H3K27ac ↑	FZD7-TWIST1-BCL2 axis via Wnt signalling	Ovarian cancer	(158)
TWIST1	Histone methylation	H3K9me3 → TWIST1 degradation	Suv39h1, Suv39h2	Gastric cancer	(159)
TP53I11	Downregulation	–	TP53I11 ↓ → AMPK, AKT/mTOR/p70S6K pathway inhibition	Breast cancer (MCF10A)	(160)
miR-204, SIRT1	miRNA silencing, Histone deacetylation	Downregulation via histone effect, HDAC activity ↑	SIRT1 ↑ → LKB1 inactivation → EMT & anoikis resistance ↑	Gastric cancer	(161)
miR-145-5p	miRNA differential expression	–	miR-145-5p ↓ in primary CRC; ↑ in metastatic CRC → anoikis resistance ↑	Colorectal cancer	(162)
SH2B3	Downstream inhibition	-	TGF-β1 ↑ → SH2B3 ↓ → JAK2/STAT3 & PI3K/AKT ↑	Lung cancer	(162)

→: Causal or regulatory relationship; ↑: Increased expression or activation; ↓: Decreased expression or inhibition.

### Part VI: Epigenetic regulation of anchorage-independent growth

Anchorage-independent growth is a hallmark of metastasis and refers to the ability of cells that have escaped anoikis to proliferate without attachment to solid surfaces (163). In ovarian cancer, malignant cells that remain free-floating within ascitic fluid accumulating in the peritoneal cavity represent a classical example of anoikis resistance coupled with anchorage-independent growth. Cytokines of the TGF-β superfamily—including bone morphogenetic proteins (BMPs), growth differentiation factors (GDFs), activins, inhibins, glial cell–derived neurotrophic factors, and nodal—provide critical regulatory signals in this process. These cytokines modulate anchorage-independent survival primarily through epigenetic regulation of SOX2 expression. Negative regulation of SOX2 by BMP2, BMP4, BMP9, TGF-β, and activin A induces anoikis via promoter methylation and SMAD1-mediated enrichment of H3K27me3. In contrast, TGF-β1 and activin A enhance SOX2 expression by increasing H3K4me3 through SMAD3, thereby promoting anoikis resistance and metastatic potential (164). Cullin 4B (CUL4B) is a scaffold protein of the Cullin 4B–RING E3 ubiquitin ligase complex, which plays a role in protein degradation, DNA damage response, and nucleotide excision repair (165). In prostate cancer, CUL4B promotes histone and DNA methylation, recruits the PRC2 complex, and increases H2AK119ub1 levels, resulting in epigenetic suppression of miR-200b/c and subsequent upregulation of BMI1 (166, 167). BMI1 is a key regulator of EMT, invasion, metastasis, cancer stem cell maintenance, and cellular senescence, and its overexpression is associated with poor prognosis. Accordingly, activation of the CUL4B–miR-200b/c–BMI1 axis enhances anchorage-independent growth and metastatic capacity in prostate cancer cells (166). Hypoxia-inducible factors (HIFs), which orchestrate cellular adaptation to hypoxic stress commonly observed in rapidly growing tumors, contribute to tumor aggressiveness (168). Prolonged hypoxia or inhibition of DNMT1 has been shown to reduce CpG methylation within the CD70 promoter, leading to increased CD70 expression. Under hypoxic conditions, DNMT1 expression is directly regulated by HIF-2α, establishing an epigenetically controlled HIF-2α–CD70 axis that promotes anchorage-independent growth and metastasis (169). Chemical and occupational carcinogens such as formaldehyde (FA) exert epigenotoxic effects by binding lysine residues on histones H3 and H4, thereby preventing acetylation and inducing DNA damage, DNA–protein crosslinks, and mutagenesis (170, 171). FA exposure reduces histone acetylation, disrupts chromatin architecture, and alters the expression of tumor suppressor genes and oncogenes. In bronchial epithelial cells, suppression of the histone variant H3.3 was shown to facilitate anchorage-independent growth in an FA-dependent manner, underscoring the carcinogenic potential of FA (171). Gliomas are primary malignant brain tumors of glial origin and are particularly prevalent in adult males (172). In glioma cells, reduced expression of miR-195 leads to derepression of cyclin D1 and cyclin E1 through their 3′UTRs, thereby promoting cell cycle progression, reducing retinoblastoma protein phosphorylation, decreasing proliferating cell nuclear antigen (PCNA) expression, and ultimately enhancing anchorage-independent growth (173). The catalytic subunit of protein phosphatase 2A (PP2Ac) suppresses DNA damage repair during chronic viral hepatitis–associated hepatocarcinogenesis. Overexpression of PP2Ac disrupts key histone modifications, including H4 arginine 3 demethylation, H4 lysine 16 acetylation, and H4 lysine 20 trimethylation, leading to dysregulated expression of genes such as IGFBP3, SOCS2, CDKN2A, FOXO1A, β-catenin, E-cadherin, D45β, and EGFR, as well as impaired DNA repair. These epigenetic alterations collectively contribute to anchorage-independent growth in HCC (174). Tissue inhibitor of metalloproteinases 1 (TIMP1), a natural inhibitor of extracellular matrix–degrading MMPs, is frequently dysregulated in cancer (174, 175). In premalignant melanocytes and melanoma cell models, increased TIMP1 expression following treatment with 5-aza-2′-deoxycytidine indicates regulation by DNA methylation. Elevated TIMP1 levels have been linked to anoikis resistance, anchorage-independent growth, and enhanced metastatic potential (176). Cervical cancer, a subset of anogenital and head and neck carcinomas, arises primarily from persistent infection with high-risk human papillomavirus (hrHPV) strains (177, 178). In hrHPV-immortalized keratinocyte cell lines, hypermethylation of tumor suppressor miRNAs—including hsa-miR-129-2, -935, -3663, -3665, and -4281—leads to their transcriptional silencing and promotes anchorage-independent growth. Treatment with the demethylating agent 2′-deoxy-5-azacytidine restores miRNA expression and reduces anchorage-independent growth, highlighting the clinical relevance of epigenetic regulation in hrHPV-associated carcinogenesis (177). Epigenetic plasticity plays a central role in enabling anchorage-independent survival and sustained proliferation of disseminated tumor cells by modulating anoikis resistance, stemness-associated transcriptional programs, and metabolic adaptation. Key epigenetic regulators involved in anchorage-independent growth are summarized in **Table 2F**.

**Table 2F:** Epigenetic regulation of extravasation, dormancy, and colonization.

Gene/Tf/Molecule	Epigenetic mechanism	Modification type	Enzyme/Complex/Pathway	Type of cancer	References
SOX2	Promoter methylation, histone modification	H3K27me3 ↑ (BMP9), H3K4me3 ↑ (TGF-β1/Activin A)	SMAD1 (BMPs), SMAD3 (TGF-β1/Activin A)	Ovarian cancer	(164)
CUL4B	Histone and DNA methylation	H2AK119ub1 ↑, miR-200b/c suppression	PRC2 recruitment → BMI1 ↑	Prostate cancer	(166, 167)
CD70	DNA demethylation	Promoter CpG hypomethylation	HIF-2α ↓ DNMT1 → CD70 ↑	Various cancers	(169)
Histone H3.3	Inhibition of histone acetylation	H3/H4 deacetylation (by FA action)	Formaldehyde → Binding to histone lysine	Bronchial epithelial cells	(171)
miR-195	miRNA downregulation	3’UTR binding ↓ (Cyclin D1/E1)	PCNA ↓, pRb phosphorylation ↓	Glioma	(173)
PP2Ac	Histone modification	H4R3me2 ↓, AcH4K16 ↓, H4K20me3 ↓	Regulation of DNA damage repair genes	Hepatocellular carcinoma	(174)
TIMP1	DNA demethylation	Expression after demethylation ↑	5-aza-2’-deoxycytidine treatment → TIMP1 ↑	Melanoma (murine model)	(176)
hsa-miR-129-2, -935, -3663, -3665, -4281	Promoter methylation	DNA hypermethylation → expression repression	2′-deoxy-5-azacytidine → methylation ↓, anchorage indep. ↓)	HPV-immortalized keratinocytes	(177)

→: Causal or regulatory relationship; ↑: Increased expression or activation; ↓: Decreased expression or inhibition.

### Part VII: Epigenetic regulation of tumor cell extravasation and colonization

During primary tumor progression, cancer cells that detach from the tumor mass enter the circulation and give rise to CTCs, which are widely regarded as precursors of metastasis (179, 180). CTCs adhere to endothelial cells of distant organs, undergo transendothelial migration through modulation of endothelial junctions, and extravasate into the surrounding tissue (179, 181). This multistep process is facilitated by adhesion molecules such as selectins, cadherins, and integrins, which mediate dynamic interactions between tumor cells and the endothelium (182). Following extravasation, disseminated tumor cells may enter a state of dormancy, characterized by prolonged cellular quiescence until microenvironmental conditions become permissive for proliferation (181). During dormancy and subsequent reactivation, tumor cells undergo extensive genetic and epigenetic reprogramming. The reversible nature of epigenetic modifications is thought to underlie the ability of dormant cells to survive hostile microenvironments and subsequently reinitiate metastatic outgrowth. While EMT facilitates intravasation, extravasation, and early dissemination, the reverse process—MET—is critical for metastatic colonization at secondary sites (10, 183–186). Because mesenchymal-like cells exhibit limited proliferative capacity, reversion to an epithelial phenotype via MET restores proliferation and enables macrometastatic growth (10). Epigenetic regulation plays a central role in EMT–MET plasticity. Studies in breast cancer cell lines have demonstrated that the coexistence of activating (H3K4me3) and repressive (H3K27me3) histone marks within the same promoter regions generates bivalent chromatin domains that suppress transcription while maintaining genes in a poised state (187). During EMT, these bivalent domains preserve transcriptional flexibility, whereas MET is associated with dynamic remodeling of these marks. Notably, expression of the H3K27me3 demethylase KDM6A decreases during EMT and is restored during MET, thereby supporting the re-expression of epithelial markers such as E-cadherin. KDM6A has also been shown to promote colonization by activating bivalent genes involved in proliferation and differentiation (187). In CRC with liver metastasis, epigenetic regulation of miRNAs contributes to phenotypic switching during colonization. In metastatic CRC tissues, miR-200c expression is reduced as a result of promoter hypermethylation, leading to upregulation of target genes such as ZEB1, ETS1, and FLT1 and reinforcing mesenchymal traits (130). In contrast, hypomethylation of the miR-200c promoter and increased miR-200c expression in primary CRC tissues and non-metastatic liver lesions are associated with elevated E-cadherin expression and reacquisition of epithelial characteristics, thereby facilitating proliferative outgrowth and macrometastasis formation (130). Global DNA methylation dynamics further contribute to metastatic competence. Comparative analyses of methylcytosine (mCyt) and hydroxymethylcytosine (hmCyt) levels revealed global hypomethylation and a significant increase in hmCyt in both primary and metastatic tissues from CRC patients with liver metastases relative to normal colon and liver tissues. However, other studies have reported reduced hmCyt levels in tumor tissues (188). Loss of hmCyt has been linked to increased DNA demethylation in human cancers (189, 190), suggesting that disrupted DNA methylation homeostasis is associated with enhanced metastatic potential and poor prognosis (191). Emerging evidence also implicates miRNAs in organ-specific colonization. miR-30b-5p expression is elevated in primary breast tumors and in cases with synchronous bone metastases, with significantly higher levels detected in bone metastases compared to metastases in other organs (192). These findings suggest that miR-30b-5p may contribute not only to metastatic progression but also to organotropism. miR-21, a well-characterized oncomiR, is upregulated across a broad spectrum of solid and hematological malignancies (193). In triple-negative breast cancer (TNBC), lysophosphatidic acid (LPA), a bioactive lipid mediator, promotes metastasis through activation of the LPA1/PI3K signaling axis, resulting in ZEB1-mediated upregulation of miR-21. In murine models, pharmacological inhibition of miR-21 or silencing of LPA1/ZEB1 significantly reduced bone colonization, underscoring the critical role of miR-21 in metastatic colonization in TNBC (194) (See **Table 2G**).

**Table 2G:** Epigenetic regulation of extravasation, dormancy, and colonization.

Gene/Tf/Molecule	Epigenetic mechanism	Modification type	Enzyme/Complex/Pathway	Type of cancer	References
KDM6A	Histone demethylation	H3K27me3 ↓ → H3K4me3 ↑ (bivalent domain dissociation)	KDM6A expression is increased by MET	Breast cancer	(187)
E-Cadherin	Transcriptional reactivation via MET	Transcriptional reactivation via MET	KDM6A, H3K27me3 ↓, H3K4me3 ↑	Breast cancer	(187)
miR-200c	Promoter methylation/hypomethylation	DNA hypermethylation or hypomethylation	ZEB1, ETS1, FLT1 targets, E-cadherin regulation	Colorectal cancer with liver metastasis	(130)
Global DNA	Global demethylation	↓ mCyt, ↓ hmCyt	–	Colon cancer and liver metastasis	(188–191)
miR-30b-5p	-	Increased expression	-	Primary breast cancer with bone metastasis	(192)
miR-21	miRNA overexpression via upstream signalling	Up-regulation by LPA-ZEB1 pathway	LPA1/PI3K/ZEB1 axis	Triple-negative breast cancer	(193, 194)

Taken together, the epigenetic mechanisms summarized in **Table 2A**-**F** illustrate that metastatic progression is governed by highly interconnected and reversible regulatory networks rather than linear genetic events. DNA methylation, histone modifications, and non-coding RNAs cooperatively regulate phenotypic plasticity across distinct stages of the metastatic cascade, from EMT initiation to colonization at distant organs. This multilayered epigenetic control provides both challenges and opportunities for therapeutic intervention, as context-dependent effects must be carefully considered in the development of precision oncology strategies.

## Epitranscriptomic regulation of EMT: the role of m6A RNA methylation

In recent years, epitranscriptomic modifications have emerged as an additional regulatory layer contributing to cancer progression and metastasis. Among these modifications, N6-methyladenosine (m^6^A) is the most abundant internal modification of eukaryotic mRNA and plays a critical role in RNA stability, splicing, nuclear export, and translation efficiency (195). Unlike DNA methylation and histone modifications, m^6^A modification directly regulates RNA stability, translation, and intracellular processing, enabling rapid and reversible modulation of gene expression programs associated with epithelial-mesenchymal plasticity (196, 197).

m^6^A regulation is coordinated by three main groups of proteins that dynamically and reversibly create, remove, and interpret N6-methyladenosine markers on RNA: “printers” (methyltransferases such as METTL3, METTL14, and WTAP), “erasers” (demethylases such as FTO and ALKBH5), and “readers” (proteins containing the YTH domain that recognize the modification) (198). There is increasing evidence that dysregulation of these regulators affects EMT-associated transcriptional networks and metastatic potential in various cancer types (196, 199).

Accumulating evidence from experimental studies indicates that aberrant m^6^A modification promotes EMT by enhancing the stability and translation of key EMT transcription factors, including SNAIL, ZEB1, and TWIST1 (200, 201). METTL3-mediated m^6^A insertion stabilizes key genes in the EMT process, thereby promoting mesenchymal phenotypes in several solid tumors; for example, METTL3, through m6A modification of ZMYM1 mRNA in gastric cancer, has increased EMT and metastasis (202). Evidence that METTL3 supports the EMT process is also supported by pathways such as MALAT1/miR-26b/HMGA2 in different tumor models, where METTL3 enhances EMT and tumor cell invasion by modifying MALAT1 with m^6^A (203). Li et al. reported that METTL3-mediated m^6^A modification affects ZEB1 mRNA and that ZEB1 expression is associated with METTL3 in hepatocellular carcinoma; these findings suggest that m^6^A modification may play a role in the regulation of ZEB1, an EMT-related transcription factor (204). ALKBH5, as the m^6^A demethylase, plays a critical role in the regulation of TGF-β-induced EMT and NSCLC metastasis. Significantly reduced ALKBH5 expression has been reported in metastatic NSCLC tissues, with ALKBH5 overexpression suppressing TGF-β-induced EMT, cell invasion, and *in vivo* metastasis, while suppression enhances these phenotypes. Mechanistically, it has been reported that removing the m6A modification of ALKBH5 reduces TGFβR2 and SMAD3 mRNA stability, increases SMAD6 expression, and thus inhibits the TGF-β/SMAD signaling pathway via YTHDF1/2/3 mediated mechanisms (205). In a separate study conducted at NSCLC, it was shown that the m^6^A demethylase ALKBH5 reduces YAP expression via YTHDF proteins and inhibits tumor growth and metastasis by suppressing YAP activity via the miR-107/LATS2 axis (206). In their study evaluating the effects of m^6^A demethylases FTO and ALKBH5 on RCC cell lines, Hu et al. found that suppression of both demethylases reduced proliferation and cell migration in ACHN, Caki-1, and 769-P cells, and led to a decrease in the expression of the EMT marker vimentin. These findings suggest that FTO and ALKBH5 support malignancy potential in RCC through EMT (207). m^6^A modification also modifies classical signaling pathways associated with EMT; for example, METTL3-dependent m6A modification contributes to EMT-related migration/invasion processes via β-catenin expression, translation, and intracellular localization (199).

Despite growing interest, the mechanistic understanding of m6A-mediated EMT regulation remains incomplete. Most studies rely on *in vitro* models, and the interplay between m6A modification and classical epigenetic mechanisms such as DNA methylation and histone modifications is still under investigation. Nevertheless, the reversible nature of m6A modification and its direct impact on EMT-associated transcripts position epitranscriptomics as a promising regulatory axis in cancer metastasis.

## Future directions

A key challenge in the field is distinguishing correlative epigenetic alterations from functionally validated drivers of metastasis. Despite substantial advances in understanding the epigenetic landscape of cancer metastasis, several critical challenges remain. One major limitation is the pronounced context dependency of epigenetic alterations, which may exert opposing effects depending on tumor type, metastatic stage, and microenvironmental cues. This complexity highlights the need for spatially and temporally resolved epigenomic analyses that capture metastatic heterogeneity at single-cell resolution.

Emerging high-resolution epigenomic technologies, including single-cell DNA methylation profiling, chromatin accessibility assays, and integrative multi-omics approaches, are expected to provide unprecedented insights into metastasis-associated epigenetic plasticity (208). In parallel, liquid biopsy–based epigenetic biomarkers, such as ctDNA methylation signatures and non-coding RNA profiles, hold considerable promise for real-time monitoring of metastatic progression, minimal residual disease, and therapeutic response (209).

From a therapeutic perspective, epigenetic alterations represent attractive yet challenging targets. While epigenetic drugs have demonstrated clinical efficacy in hematological malignancies, their application in metastatic solid tumors requires a deeper mechanistic understanding and rational combination strategies. Targeting epigenetic regulators in conjunction with conventional chemotherapy, targeted therapy, or immunotherapy may offer synergistic benefits by overcoming therapy resistance and limiting metastatic outgrowth (210).

Despite their therapeutic promise, epigenetic-targeting strategies face several limitations. These include off-target effects due to the global nature of epigenetic modifications, potential systemic toxicity, and limited specificity for cancer cells. Moreover, the dynamic and reversible nature of epigenetic regulation contributes to therapeutic resistance and adaptive reprogramming. Epigenetic plasticity may enable tumor cells to bypass targeted interventions, highlighting the need for combination therapies and more precise targeting approaches (211, 212).

In parallel with these advances, the clinical translation of epigenetic alterations is rapidly gaining attention in precision oncology. Epigenetic changes, including aberrant DNA methylation, histone modifications, and non-coding RNA dysregulation, are increasingly being explored as biomarkers for patient stratification, prognosis, and prediction of treatment response (213). For example, promoter hypermethylation of tumor suppressor genes and dysregulation of microRNAs such as the miR-200 family have been associated with metastatic potential and EMT regulation across multiple cancer types (214).

Furthermore, epigenetic-targeting agents, including DNA methyltransferase (DNMT) inhibitors and histone deacetylase (HDAC) inhibitors, are being actively investigated in clinical settings, with growing interest in their application to solid tumors (215). In addition, advances in liquid biopsy technologies now enable the detection of ctDNA methylation patterns, offering a minimally invasive approach for monitoring tumor dynamics and therapeutic response (216). Collectively, these developments highlight the strong translational potential of epigenetic regulation and support its integration into precision oncology frameworks.

Importantly, the epigenetic regulation of EMT and metastasis is highly context-dependent and influenced by tissue specificity, tumor heterogeneity, and microenvironmental factors (217). Different cancer types exhibit distinct epigenetic landscapes that shape transcriptional programs and metastatic potential. In addition, intra-tumoral heterogeneity further diversifies epigenetic states within the same tumor, while microenvironmental signals such as hypoxia, inflammation, and stromal interactions dynamically modulate epigenetic regulators. These context-dependent variations underscore the complexity of metastasis and highlight the need for tailored, cancer type–specific therapeutic strategies (212, 218).

## Conclusion

In conclusion, metastasis should be viewed as an epigenetically driven and reversible process rather than a fixed genetic endpoint. A critical yet often underappreciated aspect of metastasis is its temporal and dynamic nature. Epigenetic regulation enables reversible transitions between epithelial and mesenchymal states (EMT and MET), as well as the establishment of dormancy-associated programs that allow tumor cells to survive in a quiescent state for extended periods. These dynamic and reversible processes introduce significant challenges for therapeutic targeting, as tumor cells may shift between drug-sensitive and drug-resistant states. Consequently, epigenetic plasticity not only facilitates metastatic progression but also contributes to treatment failure and disease relapse. A comprehensive understanding of epigenetic reprogramming across distinct stages of metastatic cascade will be essential for the development of clinically actionable biomarkers and precision oncology approaches aimed at preventing or controlling metastatic disease.

Despite significant advances, several challenges remain in translating epigenetic knowledge into clinical practice. Key unresolved questions include how to distinguish driver from passenger epigenetic alterations, how to capture dynamic and reversible epigenetic states, and how to account for intra-tumoral heterogeneity. Technological limitations, including sensitivity, standardization of epigenetic assays, and integration of multi-omics data, also hinder clinical implementation.

Future research should prioritize longitudinal and single-cell epigenetic profiling, improved computational models for data integration, and the development of robust, clinically validated biomarkers. Addressing these challenges will be essential for the successful incorporation of epigenetic strategies into precision oncology.

## References

[1] Hanahan D Raw . The hallmarks of cancer. Cell. (2000) 100:57–70. doi: 10.1016/s0092-8674(00)81683-9. PMID: 10647931

[2] Hanahan D Weinberg RA . Hallmarks of cancer: the next generation. Cell. (2011) 144:646–74. doi: 10.1016/j.cell.2011.02.013. PMID: 21376230

[3] Mehlen P Puisieux A . Metastasis: a question of life or death. Nat Rev Cancer. (2006) 6:449–58. doi: 10.1038/nrc1886. PMID: 16723991

[4] Chatterjee A Rodger EJ Eccles MR . Epigenetic drivers of tumourigenesis and cancer metastasis. Semin Cancer Biol. (2018) 51:149–59. doi: 10.1016/j.semcancer.2017.08.004. PMID: 28807546

[5] Cheng PF Shakhova O Widmer DS Eichhoff OM Zingg D Frommel SC . Methylation-dependent SOX9 expression mediates invasion in human melanoma cells and is a negative prognostic factor in advanced melanoma. Genome Biol. (2015) 16:42. doi: 10.1186/s13059-015-0594-4. PMID: 25885555 PMC4378455

[6] Timp W Feinberg AP . Cancer as a dysregulated epigenome allowing cellular growth advantage at the expense of the host. Nat Rev Cancer. (2013) 13:497–510. doi: 10.1038/nrc3486. PMID: 23760024 PMC4636434

[7] Vogelstein B Papadopoulos N Velculescu VE Zhou S Diaz LA Jr Kinzler KW . Cancer genome landscapes. Science. (2013) 339:1546–58. doi: 10.1126/science.1235122. PMID: 23539594 PMC3749880

[8] Dawson MA Kouzarides T . Cancer epigenetics: from mechanism to therapy. Cell. (2012) 150:12–27. doi: 10.1016/j.cell.2012.06.013. PMID: 22770212

[9] Hanahan D . Hallmarks of cancer: new dimensions. Cancer Discov. (2022) 12:31–46. doi: 10.1158/2159-8290.cd-21-1059. PMID: 35022204

[10] Liu QL Luo M Huang C Chen HN Zhou ZG . Epigenetic regulation of epithelial to mesenchymal transition in the cancer metastatic cascade: implications for cancer therapy. Front Oncol. (2021) 11:657546. doi: 10.3389/fonc.2021.657546. PMID: 33996581 PMC8117142

[11] Bhatia S Wang P Toh A Thompson EW . New insights into the role of phenotypic plasticity and EMT in driving cancer progression. Front Mol Biosci. (2020) 7:71. doi: 10.3389/fmolb.2020.00071. PMID: 32391381 PMC7190792

[12] Swarnaliacharyya L Drw Joanmassagué . The molecular basis of cancer. In: Gray JW İsrail MA , editors.John mendelsohn PMH CBT. Philadelphia, PA, USA: Elsevier (2015).

[13] Valastyan S Weinberg RA . Tumor metastasis: molecular insights and evolving paradigms. Cell. (2011) 147:275–92. doi: 10.1016/j.cell.2011.09.024. PMID: 22000009 PMC3261217

[14] Liu M Yang J Xu B Zhang X . Tumor metastasis: mechanistic insights and therapeutic interventions. MedComm (2020). (2021) 2:587–617. doi: 10.1002/mco2.100. PMID: 34977870 PMC8706758

[15] Tan T Shi P Abbas MN Wang Y Xu J Chen Y . Epigenetic modification regulates tumor progression and metastasis through EMT (Review). Int J Oncol. (2022) 60(6):70. doi: 10.3892/ijo.2022.5360. PMID: 35445731 PMC9084613

[16] Taddei ML Giannoni E Fiaschi T Chiarugi P . Anoikis: an emerging hallmark in health and diseases. J Pathol. (2012) 226:380–93. doi: 10.1002/path.3000. PMID: 21953325

[17] Adeshakin FO Adeshakin AO Afolabi LO Yan D Zhang G Wan X . Mechanisms for modulating anoikis resistance in cancer and the relevance of metabolic reprogramming. Front Oncol. (2021) 11:626577. doi: 10.3389/fonc.2021.626577. PMID: 33854965 PMC8039382

[18] Hu X Zang X Lv Y . Detection of circulating tumor cells: advances and critical concerns. Oncol Lett. (2021) 21:422. doi: 10.3892/ol.2021.12683. PMID: 33850563 PMC8025150

[19] Lin D Shen L Luo M Zhang K Li J Yang Q . Circulating tumor cells: biology and clinical significance. Signal Transduct Target Ther. (2021) 6:404. doi: 10.1038/s41392-021-00817-8. PMID: 34803167 PMC8606574

[20] Akhmetkaliyev A Alibrahim N Shafiee D Tulchinsky E . EMT/MET plasticity in cancer and Go-or-Grow decisions in quiescence: the two sides of the same coin? Mol Cancer. (2023) 22:90. doi: 10.1186/s12943-023-01793-z. PMID: 37259089 PMC10230810

[21] Kim DJ . Molecular and immune mechanisms governing cancer metastasis, including dormancy, microenvironmental niches, and tumor-specific programs. Int J Mol Sci. (2026) 27(2):875. doi: 10.3390/ijms27020875. PMID: 41596524 PMC12840987

[22] Raghavan K Ruskin HJ Perrin D Goasmat F Burns J . Computational micromodel for epigenetic mechanisms. PloS One. (2010) 5:e14031. doi: 10.1371/journal.pone.0014031. PMID: 21152421 PMC2994705

[23] Blair LP Yan Q . Epigenetic mechanisms in commonly occurring cancers. DNA Cell Biol. (2012) 31 Suppl 1:S49–61. doi: 10.1089/dna.2012.1654. PMID: 22519822 PMC3460614

[24] Handy DE Castro R Loscalzo J . Epigenetic modifications: basic mechanisms and role in cardiovascular disease. Circulation. (2011) 123:2145–56. doi: 10.1086/675979. PMID: 21576679 PMC3107542

[25] Moore LD Le T Fan G . DNA methylation and its basic function. Neuropsychopharmacology. (2013) 38:23–38. doi: 10.1038/npp.2012.112. PMID: 22781841 PMC3521964

[26] Nishiyama A Nakanishi M . Navigating the DNA methylation landscape of cancer. Trends Genet. (2021) 37:1012–27. doi: 10.1016/j.tig.2021.05.002. PMID: 34120771

[27] Pappalardo XG Barra V . Losing DNA methylation at repetitive elements and breaking bad. Epigenet Chromatin. (2021) 14:25. doi: 10.1186/s13072-021-00400-z. PMID: 34082816 PMC8173753

[28] Ehrlich M . DNA hypomethylation in cancer cells. Epigenomics. (2009) 1:239–58. doi: 10.2217/epi.09.33. PMID: 20495664 PMC2873040

[29] Zeggar HR How-Kit A Daunay A Bettaieb I Sahbatou M Rahal K . Tumor DNA hypomethylation of LINE-1 is associated with low tumor grade of breast cancer in Tunisian patients. Oncol Lett. (2020) 20:1999–2006. doi: 10.3892/ol.2020.11745. PMID: 32724446 PMC7377197

[30] Alaskhar Alhamwe B Khalaila R Wolf J von Bulow V Harb H Alhamdan F . Histone modifications and their role in epigenetics of atopy and allergic diseases. Allergy Asthma Clin Immunol. (2018) 14:39. doi: 10.1186/s13223-018-0259-4. PMID: 29796022 PMC5966915

[31] Audia JE Campbell RM . Histone modifications and cancer. Cold Spring Harb Perspect Biol. (2016) 8:a019521. doi: 10.1101/cshperspect.a019521. PMID: 27037415 PMC4817802

[32] Bannister AJ Kouzarides T . Regulation of chromatin by histone modifications. Cell Res. (2011) 21:381–95. doi: 10.1038/cr.2011.22. PMID: 21321607 PMC3193420

[33] Gibney ER Nolan CM . Epigenetics and gene expression. Heredity (Edinb). (2010) 105:4–13. doi: 10.1038/hdy.2010.54. PMID: 20461105

[34] Kadoch C Copeland RA Keilhack H . PRC2 and SWI/SNF chromatin remodeling complexes in health and disease. Biochemistry. (2016) 55:1600–14. doi: 10.1021/acs.biochem.5b01191. PMID: 26836503

[35] Nithya Krishnamurthy SK Scott Lippman Razelle Kurzrock . Chromatin remodeling (SWI/SNF) complexes, cancer, and response to immunotherapy. In: Journal for immunoTherapy of cance London: BMJ (2022). doi: 10.1136/jitc-2022-004669

[36] Zhang Z Wang X Xin J Ding Z Liu S Fang Q . Architecture of SWI/SNF chromatin remodeling complex. Protein Cell. (2018) 9:1045–49. doi: 10.1007/s13238-018-0524-9. PMID: 29546678 PMC6251806

[37] Tang Y Wang J Lian Y Fan C Zhang P Wu Y . Linking long non-coding RNAs and SWI/SNF complexes to chromatin remodeling in cancer. Mol Cancer. (2017) 16:42. doi: 10.1186/s12943-017-0612-0. PMID: 28212646 PMC5316185

[38] Mittal P Roberts CWM . The SWI/SNF complex in cancer - biology, biomarkers and therapy. Nat Rev Clin Oncol. (2020) 17:435–48. doi: 10.1038/s41571-020-0357-3. PMID: 32303701 PMC8723792

[39] Guruprasadh Swaminathan AS Aviral Kumar Vishnu Vardhan Byroju Varsha Reddy Durgempudi Lekha Dinesh Kumar . RNA interference and nanotechnology: a promising alliance for next generation cancer therapeutics. In: Frontiers in nanotechnology Switzerland: Frontiers Media SA in Lausanne, vol. 3. (2021). doi: 10.3389/fnano.2021.694838

[40] Svoboda P . Key mechanistic principles and considerations concerning RNA interference. Front Plant Sci. (2020) 11:1237. doi: 10.3389/fpls.2020.01237. PMID: 32903622 PMC7438612

[41] Chan SH Wang LH . Regulation of cancer metastasis by microRNAs. J BioMed Sci. (2015) 22:9. doi: 10.1186/s12929-015-0113-7. PMID: 25614041 PMC4318216

[42] Maduri S . Applicability of RNA interference in cancer therapy: current status. Indian J Cancer. (2015) 52:11–21. doi: 10.4103/0019-509x.175598. PMID: 26837960

[43] Michael IP Saghafinia S Hanahan D . A set of microRNAs coordinately controls tumorigenesis, invasion, and metastasis. Proc Natl Acad Sci USA. (2019) 116:24184–95. doi: 10.1073/pnas.1913307116. PMID: 31704767 PMC6883852

[44] Liu SJ Dang HX Lim DA Feng FY Maher CA . Long noncoding RNAs in cancer metastasis. Nat Rev Cancer. (2021) 21:446–60. doi: 10.1038/s41568-021-00353-1. PMID: 33953369 PMC8288800

[45] Dawoud A Ihab Zakaria Z Hisham Rashwan H Braoudaki M Youness RA . Circular RNAs: new layer of complexity evading breast cancer heterogeneity. Non-coding RNA Res. (2023) 8:60–74. doi: 10.1016/j.ncrna.2022.09.011. PMID: 36380816 PMC9637558

[46] Shen B Wang Z Li Z Song H Ding X . Circular RNAs: an emerging landscape in tumor metastasis. Am J Cancer Res. (2019) 9:630–43. 31105992 PMC6511637

[47] Bure IV Nemtsova MV Kuznetsova EB . Histone modifications and non-coding RNAs: mutual epigenetic regulation and role in pathogenesis. Int J Mol Sci. (2022) 23(10):5801. doi: 10.3390/ijms23105801. PMID: 35628612 PMC9146199

[48] Qian S Song H Huang L Hua H Zhang X Li Z . DNA, RNA, and histone methylation regulation enzymes and their crosstalk in colorectal carcinogenesis and progression: a review of molecular mechanisms, clinical implications, and future perspectives. Cell Mol Biol Lett. (2025) 30:142. doi: 10.1186/s11658-025-00823-6. PMID: 41315942 PMC12661898

[49] Reed IKG . Mechanism and regulation of epithelial–mesenchymal transition in cancer. In: Cell health and cytoskeleton Auckland: Dove Medical Press, vol. 2015. (2015). p. 7–155—66. doi: 10.2147/chc.s73822, PMID: 30513648

[50] Huang Z Zhang Z Zhou C Liu L Huang C . Epithelial-mesenchymal transition: the history, regulatory mechanism, and cancer therapeutic opportunities. MedComm (2020). (2022) 3:e144. doi: 10.1002/mco2.144. PMID: 35601657 PMC9115588

[51] Yang J Antin P Berx G Blanpain C Brabletz T Bronner M . Guidelines and definitions for research on epithelial-mesenchymal transition. Nat Rev Mol Cell Biol. (2020) 21:341–52. doi: 10.1038/s41580-020-0237-9. PMID: 32300252 PMC7250738

[52] Mendonsa AM Na TY Gumbiner BM . E-cadherin in contact inhibition and cancer. Oncogene. (2018) 37:4769–80. doi: 10.1038/s41388-018-0304-2. PMID: 29780167 PMC6119098

[53] Cook DP Vanderhyden BC . Transcriptional census of epithelial-mesenchymal plasticity in cancer. Sci Adv. (2022) 8:eabi7640. doi: 10.1126/sciadv.abi7640. PMID: 34985957 PMC8730603

[54] Serrano-Gomez SJ Maziveyi M Alahari SK . Regulation of epithelial-mesenchymal transition through epigenetic and post-translational modifications. Mol Cancer. (2016) 15:18. doi: 10.1186/s12943-016-0502-x. PMID: 26905733 PMC4765192

[55] Kim WK Kwon Y Jang M Park M Kim J Cho S . Beta-catenin activation down-regulates cell-cell junction-related genes and induces epithelial-to-mesenchymal transition in colorectal cancers. Sci Rep. (2019) 9:18440. doi: 10.1038/s41598-019-54890-9. PMID: 31804558 PMC6895046

[56] Mylavarapu S Kumar H Kumari S Sravanthi LS Jain M Basu A . Activation of epithelial-mesenchymal transition and altered beta-catenin signaling in a novel Indian colorectal carcinoma cell line. Front Oncol. (2019) 9:54. doi: 10.3389/fonc.2019.00054. PMID: 30828563 PMC6385509

[57] Tripathi K Garg M . Mechanistic regulation of epithelial-to-mesenchymal transition through RAS signaling pathway and therapeutic implications in human cancer. J Cell Commun Signal. (2018) 12:513–27. doi: 10.1007/s12079-017-0441-3. PMID: 29330773 PMC6039341

[58] Moustakas A Heldin CH . Mechanisms of TGFbeta-induced epithelial-mesenchymal transition. J Clin Med. (2016) 5():63. doi: 10.3390/jcm5070063. PMID: 27367735 PMC4961994

[59] Hua W Ten Dijke P Kostidis S Giera M Hornsveld M . TGFbeta-induced metabolic reprogramming during epithelial-to-mesenchymal transition in cancer. Cell Mol Life Sci. (2020) 77:2103–23. doi: 10.1007/s00018-019-03398-6. PMID: 31822964 PMC7256023

[60] Zhang J Zheng G Zhou L Li P Yun M Shi Q . Notch signalling induces epithelial mesenchymal transition to promote metastasis in oral squamous cell carcinoma. Int J Mol Med. (2018) 42:2276–84. doi: 10.3892/ijmm.2018.3769. PMID: 30015856

[61] Zhou B Lin W Long Y Yang Y Zhang H Wu K . Notch signaling pathway: architecture, disease, and therapeutics. Signal Transduct Target Ther. (2022) 7:95. doi: 10.1038/s41392-022-00934-y. PMID: 35332121 PMC8948217

[62] Tam SY Wu VWC Law HKW . Hypoxia-induced epithelial-mesenchymal transition in cancers: HIF-1alpha and beyond. Front Oncol. (2020) 10:486. doi: 10.3389/fonc.2020.00486. PMID: 32322559 PMC7156534

[63] Zheng HC . The molecular mechanisms of chemoresistance in cancers. Oncotarget. (2017) 8:59950–64. doi: 10.18632/oncotarget.19048. PMID: 28938696 PMC5601792

[64] Li LC . Epigenetics of prostate cancer. Front Biosci. (2007) 12:3377–97. doi: 10.2741/2320. PMID: 17485307

[65] Uysal F Ozturk S Akkoyunlu G . DNMT1, DNMT3A and DNMT3B proteins are differently expressed in mouse oocytes and early embryos. J Mol Histol. (2017) 48:417–26. doi: 10.1007/s10735-017-9739-y. PMID: 29027601

[66] Casalino L Verde P . Multifaceted roles of DNA methylation in neoplastic transformation, from tumor suppressors to EMT and metastasis. Genes (Basel). (2020) 11(8):922. doi: 10.3390/genes11080922. PMID: 32806509 PMC7463745

[67] Chen LH Hsu WL Tseng YJ Liu DW Weng CF . Involvement of DNMT 3B promotes epithelial-mesenchymal transition and gene expression profile of invasive head and neck squamous cell carcinomas cell lines. BMC Cancer. (2016) 16:431. doi: 10.1186/s12885-016-2468-x. PMID: 27391030 PMC4938990

[68] Brambert PR Kelpsch DJ Hameed R Desai CV Calafiore G Godley LA . DNMT3B7 expression promotes tumor progression to a more aggressive phenotype in breast cancer cells. PloS One. (2015) 10:e0117310. doi: 10.1371/journal.pone.0117310. PMID: 25607950 PMC4301645

[69] Ostler KR Davis EM Payne SL Gosalia BB Exposito-Cespedes J Le Beau MM . Cancer cells express aberrant DNMT3B transcripts encoding truncated proteins. Oncogene. (2007) 26:5553–63. doi: 10.1038/sj.onc.1210351. PMID: 17353906 PMC2435620

[70] Roll JD Rivenbark AG Jones WD Coleman WB . DNMT3b overexpression contributes to a hypermethylator phenotype in human breast cancer cell lines. Mol Cancer. (2008) 7:15. doi: 10.1186/1476-4598-7-15. PMID: 18221536 PMC2246151

[71] Cardenas H Vieth E Lee J Segar M Liu Y Nephew KP . TGF-beta induces global changes in DNA methylation during the epithelial-to-mesenchymal transition in ovarian cancer cells. Epigenetics. (2014) 9:1461–72. doi: 10.4161/15592294.2014.971608. PMID: 25470663 PMC4622747

[72] Mehta A Dobersch S Romero-Olmedo AJ Barreto G . Epigenetics in lung cancer diagnosis and therapy. Cancer Metastasis Rev. (2015) 34:229–41. doi: 10.1007/s10555-015-9563-3. PMID: 25939322

[73] Zhang N Liu Y Wang Y Zhao M Tu L Luo F . Decitabine reverses TGF-beta1-induced epithelial-mesenchymal transition in non-small-cell lung cancer by regulating miR-200/ZEB axis. Drug Des Devel Ther. (2017) 11:969–83. doi: 10.2147/dddt.s129305. PMID: 28405157 PMC5378468

[74] Du Q Luu PL Stirzaker C Clark SJ . Methyl-CpG-binding domain proteins: readers of the epigenome. Epigenomics. (2015) 7:1051–73. doi: 10.2217/epi.15.39. PMID: 25927341

[75] Angulo JC Manini C Lopez JI Pueyo A Colas B Ropero S . The role of epigenetics in the progression of clear cell renal cell carcinoma and the basis for future epigenetic treatments. Cancers (Basel). (2021) 13(9):2071. doi: 10.3390/cancers13092071. PMID: 33922974 PMC8123355

[76] Li L Li N Liu N Huo F Zheng J . MBD2 correlates with a poor prognosis and tumor progression in renal cell carcinoma. Onco Targets Ther. (2020) 13:10001–12. doi: 10.1016/j.prp.2023.154886. PMID: 33116585 PMC7548338

[77] Chen R Gan Q Zhao S Zhang D Wang S Yao L . DNA methylation of miR-138 regulates cell proliferation and EMT in cervical cancer by targeting EZH2. BMC Cancer. (2022) 22:488. doi: 10.1186/s12885-022-09477-5. PMID: 35505294 PMC9063191

[78] Chen JF Yan Q . The roles of epigenetics in cancer progression and metastasis. Biochem J. (2021) 478:3373–93. doi: 10.1042/bcj20210084. PMID: 34520519 PMC8524384

[79] Markouli M Strepkos D Basdra EK Papavassiliou AG Piperi C . Prominent role of histone modifications in the regulation of tumor metastasis. Int J Mol Sci. (2021) 22(5):2778. doi: 10.3390/ijms22052778. PMID: 33803458 PMC7967218

[80] Zhao Z Shilatifard A . Epigenetic modifications of histones in cancer. Genome Biol. (2019) 20:245. doi: 10.1186/s13059-019-1870-5. PMID: 31747960 PMC6868810

[81] Kong F Ma L Wang X You H Zheng K Tang R . Regulation of epithelial-mesenchymal transition by protein lysine acetylation. Cell Commun Signal. (2022) 20:57. doi: 10.1186/s12964-022-00870-y. PMID: 35484625 PMC9052664

[82] Boulding T McCuaig RD Tan A Hardy K Wu F Dunn J . LSD1 activation promotes inducible EMT programs and modulates the tumour microenvironment in breast cancer. Sci Rep. (2018) 8:73. doi: 10.1038/s41598-017-17913-x. PMID: 29311580 PMC5758711

[83] Lin T Ponn A Hu X Law BK Lu J . Requirement of the histone demethylase LSD1 in Snai1-mediated transcriptional repression during epithelial-mesenchymal transition. Oncogene. (2010) 29:4896–904. doi: 10.1038/onc.2010.234. PMID: 20562920 PMC3093107

[84] Wanna-Udom S Terashima M Suphakhong K Ishimura A Takino T Suzuki T . KDM2B is involved in the epigenetic regulation of TGF-beta-induced epithelial-mesenchymal transition in lung and pancreatic cancer cell lines. J Biol Chem. (2021) 296:100213. doi: 10.1074/jbc.ra120.015502. PMID: 33779563 PMC7948487

[85] Yi X Guo J Guo J Sun S Yang P Wang J . EZH2-mediated epigenetic silencing of TIMP2 promotes ovarian cancer migration and invasion. Sci Rep. (2017) 7:3568. doi: 10.1038/s41598-017-03362-z. PMID: 28620234 PMC5472630

[86] Wang R Liu J Li K Yang G Chen S Wu J . An SETD1A/Wnt/beta-catenin feedback loop promotes NSCLC development. J Exp Clin Cancer Res. (2021) 40:318. doi: 10.1186/s13046-021-02119-x. PMID: 34645486 PMC8513302

[87] Wang S Bleeck A Nadif Kasri N Kleefstra T van Rhijn JR Schubert D . SETD1A mediated H3K4 methylation and its role in neurodevelopmental and neuropsychiatric disorders. Front Mol Neurosci. (2021) 14:772000. doi: 10.3389/fnmol.2021.772000. PMID: 34803610 PMC8595121

[88] Wu J Chai H Shan H Pan C Xu X Dong W . Histone methyltransferase SETD1A induces epithelial-mesenchymal transition to promote invasion and metastasis through epigenetic reprogramming of Snail in gastric cancer. Front Cell Dev Biol. (2021) 9:657888. doi: 10.3389/fcell.2021.657888. PMID: 34164392 PMC8215546

[89] Patel MM Adrada BE . Hereditary breast cancer: BRCA mutations and beyond. Radiol Clin North Am. (2024) 62:627–42. doi: 10.1016/j.rcl.2023.12.014, PMID: 38777539

[90] Du M Gong P Zhang Y Liu Y Liu X Zhang F . Histone methyltransferase SETD1A participates in lung cancer progression. Thorac Cancer. (2021) 12:2247–57. doi: 10.21203/rs.3.rs-66780/v1. PMID: 34219384 PMC8365002

[91] Lee YM Kim SH Kim MS Kim DC Lee EH Lee JS . Epigenetic role of histone lysine methyltransferase and demethylase on the expression of transcription factors associated with the epithelial-to-mesenchymal transition of lung adenocarcinoma metastasis to the brain. Cancers (Basel). (2020) 12(12):3632. doi: 10.3390/cancers12123632. PMID: 33291558 PMC7761791

[92] Hu Y Nie Q Dai M Chen F Wu H . Histone deacetylases inhibit the Snail2-mediated EMT during metastasis of hepatocellular carcinoma cells. Front Cell Dev Biol. (2020) 8:752. doi: 10.3389/fcell.2020.00752. PMID: 32850856 PMC7419474

[93] Hu Y Zheng Y Dai M Wang X Wu J Yu B . G9a and histone deacetylases are crucial for Snail2-mediated E-cadherin repression and metastasis in hepatocellular carcinoma. Cancer Sci. (2019) 110:3442–52. doi: 10.1111/cas.14173. PMID: 31432592 PMC6825017

[94] Hu Y Zheng Y Dai M Wu J Yu B Zhang H . Snail2 induced E-cadherin suppression and metastasis in lung carcinoma facilitated by G9a and HDACs. Cell Adh Migr. (2019) 13:285–92. doi: 10.1080/19336918.2019.1638689. PMID: 31271097 PMC6629185

[95] Song Y Wang R Li LW Liu X Wang YF Wang QX . Long non-coding RNA HOTAIR mediates the switching of histone H3 lysine 27 acetylation to methylation to promote epithelial-to-mesenchymal transition in gastric cancer. Int J Oncol. (2019) 54:77–86. doi: 10.3892/ijo.2018.4625. PMID: 30431069 PMC6254860

[96] Grossi E Raimondi I Goni E Gonzalez J Marchese FP Chapaprieta V . A lncRNA-SWI/SNF complex crosstalk controls transcriptional activation at specific promoter regions. Nat Commun. (2020) 11:936. doi: 10.1038/s41467-020-14623-3. PMID: 32071317 PMC7028943

[97] Chava S Bugide S Edwards YJK Gupta R . Disruptor of telomeric silencing 1-like promotes ovarian cancer tumor growth by stimulating pro-tumorigenic metabolic pathways and blocking apoptosis. Oncogenesis. (2021) 10:48. doi: 10.1038/s41389-021-00339-6. PMID: 34253709 PMC8275629

[98] Cho MH Park JH Choi HJ Park MK Won HY Park YJ . DOT1L cooperates with the c-Myc-p300 complex to epigenetically derepress CDH1 transcription factors in breast cancer progression. Nat Commun. (2015) 6:7821. doi: 10.1038/ncomms8821. PMID: 26199140 PMC4525167

[99] Song Y Li ZX Liu X Wang R Li LW Zhang Q . The Wnt/beta-catenin and PI3K/Akt signaling pathways promote EMT in gastric cancer by epigenetic regulation via H3 lysine 27 acetylation. Tumour Biol. (2017) 39:1010428317712617. doi: 10.1177/1010428317712617. PMID: 28671020

[100] Rui Chang PZ Jiacong You . Post-translational modifications of EMT transcriptional factors in cancer metastasis. Open Life Sci. (2016) 11:237–43. doi: 10.1515/biol-2016-0033. PMID: 31755547

[101] Xu R Won JY Kim CH Kim DE Yim H . Roles of the phosphorylation of transcriptional factors in epithelial-mesenchymal transition. J Oncol. (2019) 2019:5810465. doi: 10.1155/2019/5810465. PMID: 31275381 PMC6582791

[102] Du C Zhang C Hassan S Biswas MH Balaji KC . Protein kinase D1 suppresses epithelial-to-mesenchymal transition through phosphorylation of snail. Cancer Res. (2010) 70:7810–9. doi: 10.1158/0008-5472.can-09-4481. PMID: 20940406

[103] Dong B Wu Y . Epigenetic regulation and post-translational modifications of SNAI1 in cancer metastasis. Int J Mol Sci. (2021) 22(20):11062. doi: 10.3390/ijms222011062. PMID: 34681726 PMC8538584

[104] Kaneko T Dehari H Sasaki T Igarashi T Ogi K Okamoto JY . Hypoxia-induced epithelial-mesenchymal transition is regulated by phosphorylation of GSK3-beta via PI3 K/Akt signaling in oral squamous cell carcinoma. Oral Surg Oral Med Oral Pathol Oral Radiol. (2016) 122:719–30. doi: 10.1016/j.oooo.2016.06.008. PMID: 27614812

[105] Zheng H Li W Wang Y Liu Z Cai Y Xie T . Glycogen synthase kinase-3 beta regulates Snail and beta-catenin expression during Fas-induced epithelial-mesenchymal transition in gastrointestinal cancer. Eur J Cancer. (2013) 49:2734–46. doi: 10.1016/j.ejca.2013.03.014. PMID: 23582741

[106] Gajula RP Chettiar ST Williams RD Nugent K Kato Y Wang H . Structure-function studies of the bHLH phosphorylation domain of TWIST1 in prostate cancer cells. Neoplasia. (2015) 17:16–31. doi: 10.1016/j.neo.2014.10.009. PMID: 25622896 PMC4309734

[107] Bulatov E Valiullina A Sayarova R Rizvanov A . Promising new therapeutic targets for regulation of inflammation and immunity: RING-type E3 ubiquitin ligases. Immunol Lett. (2018) 202:44–51. doi: 10.1016/j.imlet.2018.08.001. PMID: 30099009

[108] Chauhan AK Sun Y Zhu Q Wani AA . Timely upstream events regulating nucleotide excision repair by ubiquitin-proteasome system: ubiquitin guides the way. DNA Repair (Amst). (2021) 103:103128. doi: 10.1016/j.dnarep.2021.103128. PMID: 33991872 PMC8206018

[109] Dong X Han Y Zhang E Wang Y Zhang P Wang C . Tumor suppressor DCAF15 inhibits epithelial-mesenchymal transition by targeting ZEB1 for proteasomal degradation in hepatocellular carcinoma. Aging (Albany NY). (2021) 13:10603–18. doi: 10.18632/aging.202823. PMID: 33833131 PMC8064142

[110] Zheng J Shi Z Yang P Zhao Y Tang W Ye S . ERK-Smurf1-RhoA signaling is critical for TGFbeta-drived EMT and tumor metastasis. Life Sci Alliance. (2022) 5(10):e202101330. doi: 10.26508/lsa.202101330. PMID: 35654587 PMC9163791

[111] Li X Yuan J Song C Lei Y Xu J Zhang G . Deubiquitinase USP39 and E3 ligase TRIM26 balance the level of ZEB1 ubiquitination and thereby determine the progression of hepatocellular carcinoma. Cell Death Differ. (2021) 28:2315–32. doi: 10.1038/s41418-021-00754-7. PMID: 33649471 PMC8329202

[112] Wu L Zhao N Zhou Z Chen J Han S Zhang X . PLAGL2 promotes the proliferation and migration of gastric cancer cells via USP37-mediated deubiquitination of Snail1. Theranostics. (2021) 11:700–14. doi: 10.7150/thno.47800. PMID: 33391500 PMC7738862

[113] Yang-Hartwich Y Tedja R Roberts CM Goodner-Bingham J Cardenas C Gurea M . p53-Pirh2 complex promotes Twist1 degradation and inhibits EMT. Mol Cancer Res. (2019) 17:153–64. doi: 10.1158/1541-7786.mcr-18-0238. PMID: 30131448 PMC6800184

[114] Chanda A Sarkar A Bonni S . The SUMO system and TGFbeta signaling interplay in regulation of epithelial-mesenchymal transition: Implications for cancer progression. Cancers (Basel). (2018) 10(8):264. doi: 10.3390/cancers10080264. PMID: 30096838 PMC6115711

[115] Kroonen JS Vertegaal ACO . Targeting SUMO signaling to wrestle cancer. Trends Cancer. (2021) 7:496–510. doi: 10.1016/j.trecan.2020.11.009. PMID: 33353838

[116] Zhang X Wang H Wang H Xiao F Seth P Xu W . SUMO-specific cysteine protease 1 promotes epithelial mesenchymal transition of prostate cancer cells via regulating SMAD4 deSUMOylation. Int J Mol Sci. (2017) 18(4):808. doi: 10.3390/ijms18040808. PMID: 28417919 PMC5412392

[117] Ha HL Kwon T Bak IS Erikson RL Kim BY Yu DY . IGF-II induced by hepatitis B virus X protein regulates EMT via SUMO mediated loss of E-cadherin in mice. Oncotarget. (2016) 7:56944–57. doi: 10.18632/oncotarget.10922. PMID: 27486970 PMC5302964

[118] Ren YH Liu KJ Wang M Yu YN Yang K Chen Q . De-SUMOylation of FOXC2 by SENP3 promotes the epithelial-mesenchymal transition in gastric cancer cells. Oncotarget. (2014) 5:7093–104. doi: 10.18632/oncotarget.2197. PMID: 25216525 PMC4196186

[119] Bouyssou JM Manier S Huynh D Issa S Roccaro AM Ghobrial IM . Regulation of microRNAs in cancer metastasis. Biochim Biophys Acta. (2014) 1845:255–65. doi: 10.1016/j.bbcan.2014.02.002. PMID: 24569228 PMC4170185

[120] Annese T Tamma R De Giorgis M Ribatti D . microRNAs biogenesis, functions and role in tumor angiogenesis. Front Oncol. (2020) 10:581007. doi: 10.3389/fonc.2020.581007. PMID: 33330058 PMC7729128

[121] Sundararajan V Burk UC Bajdak-Rusinek K . Revisiting the miR-200 family: A clan of five siblings with essential roles in development and disease. Biomolecules. (2022) 12(6):781. doi: 10.3390/biom12060781. PMID: 35740906 PMC9221129

[122] O'Brien SJ Carter JV Burton JF Oxford BG Schmidt MN Hallion JC . The role of the miR-200 family in epithelial-mesenchymal transition in colorectal cancer: a systematic review. Int J Cancer. (2018) 142:2501–11. doi: 10.1002/ijc.31282, PMID: 29388209

[123] Frixa T Sacconi A Cioce M Roscilli G Ferrara FF Aurisicchio L . MicroRNA-128-3p-mediated depletion of Drosha promotes lung cancer cell migration. Carcinogenesis. (2018) 39:293–304. doi: 10.1093/carcin/bgx134. PMID: 29236960

[124] Su F Zhao J Qin S Wang R Li Y Wang Q . Over-expression of Thrombospondin 4 correlates with loss of miR-142 and contributes to migration and vascular invasion of advanced hepatocellular carcinoma. Oncotarget. (2017) 8:23277–88. doi: 10.18632/oncotarget.15054. PMID: 28177895 PMC5410303

[125] Yu Q Xiang L Yin L Liu X Yang D Zhou J . Loss-of-function of miR-142 by hypermethylation promotes TGF-beta-mediated tumour growth and metastasis in hepatocellular carcinoma. Cell Prolif. (2017) 50(6):e12384. doi: 10.1111/cpr.12384, PMID: 28963738 PMC6529086

[126] Sun L Liang J Wang Q Li Z Du Y Xu X . MicroRNA-137 suppresses tongue squamous carcinoma cell proliferation, migration and invasion. Cell Prolif. (2016) 49:628–35. doi: 10.1111/cpr.12287. PMID: 27571935 PMC6495205

[127] Chen Z Wang X Liu R Chen L Yi J Qi B . KDM4B-mediated epigenetic silencing of miRNA-615-5p augments RAB24 to facilitate Malignancy of hepatoma cells. Oncotarget. (2017) 8:17712–25. doi: 10.18632/oncotarget.10832. PMID: 27487123 PMC5392280

[128] Neves R Scheel C Weinhold S Honisch E Iwaniuk KM Trompeter HI . Role of DNA methylation in miR-200c/141 cluster silencing in invasive breast cancer cells. BMC Res Notes. (2010) 3:219. doi: 10.1186/1756-0500-3-219. PMID: 20682048 PMC3161370

[129] Ding X Park SI McCauley LK Wang CY . Signaling between transforming growth factor beta (TGF-beta) and transcription factor SNAI2 represses expression of microRNA miR-203 to promote epithelial-mesenchymal transition and tumor metastasis. J Biol Chem. (2013) 288:10241–53. doi: 10.1074/jbc.m112.443655. PMID: 23447531 PMC3624408

[130] Hur K Toiyama Y Takahashi M Balaguer F Nagasaka T Koike J . MicroRNA-200c modulates epithelial-to-mesenchymal transition (EMT) in human colorectal cancer metastasis. Gut. (2013) 62:1315–26. doi: 10.1136/gutjnl-2011-301846. PMID: 22735571 PMC3787864

[131] Chen J Gao S Wang C Wang Z Zhang H Huang K . Pathologically decreased expression of miR-193a contributes to metastasis by targeting WT1-E-cadherin axis in non-small cell lung cancers. J Exp Clin Cancer Res. (2016) 35:173. doi: 10.1186/s13046-016-0450-8. PMID: 27821145 PMC5100283

[132] Dhayat SA Traeger MM Rehkaemper J Stroese AJ Steinestel K Wardelmann E . Clinical impact of epithelial-to-mesenchymal transition regulating microRNAs in pancreatic ductal adenocarcinoma. Cancers (Basel). (2018) 10(9):328. doi: 10.3390/cancers10090328. PMID: 30217058 PMC6162771

[133] Bhagirath D Yang TL Akoto T Patel N Tabatabai LZ Saini S . MicroRNA-4287 is a novel tumor suppressor microRNA controlling epithelial-to mesenchymal transition in prostate cancer. Oncotarget. (2020) 11:4681–92. doi: 10.18632/oncotarget.27849. PMID: 33473254 PMC7771715

[134] Dhamija S Diederichs S . From junk to master regulators of invasion: lncRNA functions in migration, EMT and metastasis. Int J Cancer. (2016) 139:269–80. doi: 10.1002/ijc.30039. PMID: 26875870

[135] Heery R Finn SP Cuffe S Gray SG . Long non-coding RNAs: Key regulators of epithelial-mesenchymal transition, tumour drug resistance and cancer stem cells. Cancers (Basel). (2017) 9(4):38. doi: 10.3390/cancers9040038. PMID: 28430163 PMC5406713

[136] Hamad H Othman G Kardagh R . Investigating the role of HOTAIR and MALAT1 long noncoding RNAs and their relations with bone marrow environment in acute myeloid leukemia subtypes: Biomarkers and treatment response. Adv Hematol. (2025) 2025:3459924. doi: 10.1155/ah/3459924. PMID: 41112951 PMC12530475

[137] Statello L Guo CJ Chen LL Huarte M . Gene regulation by long non-coding RNAs and its biological functions. Nat Rev Mol Cell Biol. (2021) 22:96–118. doi: 10.1038/s41580-020-00315-9. PMID: 33353982 PMC7754182

[138] Bure IV Nemtsova MV Zaletaev DV . Roles of E-cadherin and noncoding RNAs in the epithelial-mesenchymal transition and progression in gastric cancer. Int J Mol Sci. (2019) 20(12):2870. doi: 10.3390/ijms20122870. PMID: 31212809 PMC6627057

[139] Entezari M Taheriazam A Orouei S Fallah S Sanaei A Hejazi ES . LncRNA-miRNA axis in tumor progression and therapy response: An emphasis on molecular interactions and therapeutic interventions. BioMed Pharmacother. (2022) 154:113609. doi: 10.1016/j.biopha.2022.113609. PMID: 36037786

[140] Eptaminitaki GC Stellas D Bonavida B Baritaki S . Long non-coding RNAs (lncRNAs) signaling in cancer chemoresistance: From prediction to druggability. Drug Resist Update. (2022) 65:100866. doi: 10.1016/j.drup.2022.100866. PMID: 36198236

[141] Guo W Liu GM Guan JY Chen YJ Zhao YZ Wang K . Epigenetic regulation of cutaneous T-cell lymphoma is mediated by dysregulated lncRNA MALAT1 through modulation of tumor microenvironment. Front Oncol. (2022) 12:977266. doi: 10.3389/fonc.2022.977266. PMID: 36059695 PMC9433805

[142] Liu F Ma X Bian X Zhang C Liu X Liu Q . LINC00586 represses ASXL1 expression thus inducing epithelial-to-mesenchymal transition of colorectal cancer cells through LSD1-mediated H3K4me2 demethylation. Front Pharmacol. (2022) 13:887822. doi: 10.3389/fphar.2022.887822. PMID: 35586041 PMC9108668

[143] Huang CS Tsai CH Yu CP Wu YS Yee MF Ho JY . Long noncoding RNA LINC02470 sponges microRNA-143-3p and enhances SMAD3-mediated epithelial-to-mesenchymal transition to promote the aggressive properties of bladder cancer. Cancers (Basel). (2022) 14(4):968. doi: 10.3390/cancers14040968. PMID: 35205713 PMC8870681

[144] Hu XT Xing W Zhao RS Tan Y Wu XF Ao LQ . HDAC2 inhibits EMT-mediated cancer metastasis by downregulating the long noncoding RNA H19 in colorectal cancer. J Exp Clin Cancer Res. (2020) 39:270. doi: 10.1186/s13046-020-01783-9. PMID: 33267897 PMC7709355

[145] Kaneko S Bonasio R Saldana-Meyer R Yoshida T Son J Nishino K . Interactions between JARID2 and noncoding RNAs regulate PRC2 recruitment to chromatin. Mol Cell. (2014) 53:290–305. doi: 10.1016/j.molcel.2013.11.012. PMID: 24374312 PMC4026005

[146] Tange S Oktyabri D Terashima M Ishimura A Suzuki T . JARID2 is involved in transforming growth factor-beta-induced epithelial-mesenchymal transition of lung and colon cancer cell lines. PloS One. (2014) 9:e115684. doi: 10.1371/journal.pone.0115684. PMID: 25542019 PMC4277293

[147] Terashima M Tange S Ishimura A Suzuki T . MEG3 long noncoding RNA contributes to the epigenetic regulation of epithelial-mesenchymal transition in lung cancer cell lines. J Biol Chem. (2017) 292:82–99. doi: 10.1074/jbc.m116.750950. PMID: 27852821 PMC5217702

[148] Peng L Jiang B Yuan X Qiu Y Peng J Huang Y . Super-enhancer-associated long noncoding RNA HCCL5 is activated by ZEB1 and promotes the Malignancy of hepatocellular carcinoma. Cancer Res. (2019) 79:572–84. doi: 10.1158/0008-5472.can-18-0367. PMID: 30482773

[149] Hao F Zhang Y Hou J Zhao B . Chromatin remodeling and cancer: the critical influence of the SWI/SNF complex. Epigenet Chromatin. (2025) 18:22. doi: 10.1186/s13072-025-00590-w. PMID: 40269969 PMC12016160

[150] Liu L Zhang Y Lu J . The roles of long noncoding RNAs in breast cancer metastasis. Cell Death Dis. (2020) 11:749. doi: 10.1038/s41419-020-02954-4. PMID: 32929060 PMC7490374

[151] Wu W Chen F Cui X Yang L Chen J Zhao J . LncRNA NKILA suppresses TGF-beta-induced epithelial-mesenchymal transition by blocking NF-kappaB signaling in breast cancer. Int J Cancer. (2018) 143:2213–24. doi: 10.3390/jcm5020013. PMID: 29761481

[152] Frisch SM Schaller M Cieply B . Mechanisms that link the oncogenic epithelial-mesenchymal transition to suppression of anoikis. J Cell Sci. (2013) 126:21–9. doi: 10.1016/s0955-0674(00)00251-9. PMID: 23516327 PMC3603508

[153] Paoli P Giannoni E Chiarugi P . Anoikis molecular pathways and its role in cancer progression. Biochim Biophys Acta. (2013) 1833:3481–98. doi: 10.1016/j.bbamcr.2013.06.026. PMID: 23830918

[154] Cao Z Livas T Kyprianou N . Anoikis and EMT: Lethal "liaisons" during cancer progression. Crit Rev Oncog. (2016) 21:155–68. doi: 10.1615/critrevoncog.2016016955. PMID: 27915969 PMC5451151

[155] Khan SU Fatima K Malik F . Understanding the cell survival mechanism of anoikis-resistant cancer cells during different steps of metastasis. Clin Exp Metastasis. (2022) 39:715–26. doi: 10.1007/s10585-022-10172-9. PMID: 35829806

[156] Liu W Vivian CJ Brinker AE Hampton KR Lianidou E Welch DR . Microenvironmental influences on metastasis suppressor expression and function during a metastatic cell's journey. Cancer Microenviron. (2014) 7:117–31. doi: 10.1007/s12307-014-0148-4. PMID: 24938990 PMC4275500

[157] Bao W Qiu H Yang T Luo X Zhang H Wan X . Upregulation of TrkB promotes epithelial-mesenchymal transition and anoikis resistance in endometrial carcinoma. PloS One. (2013) 8:e70616. doi: 10.1371/journal.pone.0070616. PMID: 23936232 PMC3728299

[158] Tan M Asad M Heong V Wong MK Tan TZ Ye J . The FZD7-TWIST1 axis is responsible for anoikis resistance and tumorigenesis in ovarian carcinoma. Mol Oncol. (2019) 13:757–80. doi: 10.1002/1878-0261.12425. PMID: 30548372 PMC6441896

[159] Sakamoto A Akiyama Y Shimada S Zhu WG Yuasa Y Tanaka S . DNA methylation in the exon 1 region and complex regulation of Twist1 expression in gastric cancer cells. PloS One. (2015) 10:e0145630. doi: 10.1371/journal.pone.0145630. PMID: 26695186 PMC4687923

[160] Xiao T Xu Z Zhang H Geng J Qiao Y Liang Y . TP53I11 suppresses epithelial-mesenchymal transition and metastasis of breast cancer cells. BMB Rep. (2019) 52:379–84. doi: 10.5483/bmbrep.2019.52.6.173. PMID: 30940320 PMC6605526

[161] Zhang L Wang X Chen P . MiR-204 down regulates SIRT1 and reverts SIRT1-induced epithelial-mesenchymal transition, anoikis resistance and invasion in gastric cancer cells. BMC Cancer. (2013) 13:290. doi: 10.1186/1471-2407-13-290. PMID: 23768087 PMC3710153

[162] Cheng X Shen T Liu P Fang S Yang Z Li Y . mir-145-5p is a suppressor of colorectal cancer at early stage, while promotes colorectal cancer metastasis at late stage through regulating AKT signaling evoked EMT-mediated anoikis. BMC Cancer. (2022) 22:1151. doi: 10.1186/s12885-022-10182-6. PMID: 36348305 PMC9644492

[163] Mori S Chang JT Andrechek ER Matsumura N Baba T Yao G . Anchorage-independent cell growth signature identifies tumors with metastatic potential. Oncogene. (2009) 28:2796–805. doi: 10.1038/onc.2009.139. PMID: 19483725 PMC3008357

[164] Shonibare Z Monavarian M O'Connell K Altomare D Shelton A Mehta S . Reciprocal SOX2 regulation by SMAD1-SMAD3 is critical for anoikis resistance and metastasis in cancer. Cell Rep. (2022) 40:111066. doi: 10.1016/j.celrep.2022.111066. PMID: 35905726 PMC9899501

[165] Londin ER Adijanto J Philp N Novelli A Vitale E Perria C . Donor splice-site mutation in CUL4B is likely cause of X-linked intellectual disability. Am J Med Genet A. (2014) 164A:2294–9. doi: 10.1002/ajmg.a.36629. PMID: 24898194 PMC4404493

[166] Jiao M Qi M Zhang F Hu J Feng T Zhao M . CUL4B regulates cancer stem-like traits of prostate cancer cells by targeting BMI1 via miR200b/c. Prostate. (2019) 79:1294–303. doi: 10.1002/pros.23835. PMID: 31111526

[167] Srinivasan M Bharali DJ Sudha T Khedr M Guest I Sell S . Downregulation of Bmi1 in breast cancer stem cells suppresses tumor growth and proliferation. Oncotarget. (2017) 8:38731–42. doi: 10.18632/oncotarget.16317. PMID: 28418883 PMC5503567

[168] Keith B Johnson RS Simon MC . HIF1alpha and HIF2alpha: sibling rivalry in hypoxic tumour growth and progression. Nat Rev Cancer. (2011) 12:9–22. doi: 10.1038/nrc3183. PMID: 22169972 PMC3401912

[169] Kitajima S Lee KL Fujioka M Sun W You J Chia GS . Hypoxia-inducible factor-2 alpha up-regulates CD70 under hypoxia and enhances anchorage-independent growth and aggressiveness in cancer cells. Oncotarget. (2018) 9:19123–35. doi: 10.18632/oncotarget.24919. PMID: 29721188 PMC5922382

[170] Chen D Fang L Mei S Li H Xu X Des Marais TL . Regulation of chromatin assembly and cell transformation by formaldehyde exposure in human cells. Environ Health Perspect. (2017) 125:097019. doi: 10.1289/ehp1275. PMID: 28937961 PMC5915180

[171] Swenberg JA Moeller BC Lu K Rager JE Fry RC Starr TB . Formaldehyde carcinogenicity research: 30 years and counting for mode of action, epidemiology, and cancer risk assessment. Toxicol Pathol. (2013) 41:181–99. doi: 10.1016/b978-0-12-415759-0.00031-5. PMID: 23160431 PMC3893912

[172] Yang K Wu Z Zhang H Zhang N Wu W Wang Z . Glioma targeted therapy: insight into future of molecular approaches. Mol Cancer. (2022) 21:39. doi: 10.1186/s12943-022-01513-z. PMID: 35135556 PMC8822752

[173] Hui W Yuntao L Lun L WenSheng L ChaoFeng L HaiYong H . MicroRNA-195 inhibits the proliferation of human glioma cells by directly targeting cyclin D1 and cyclin E1. PloS One. (2013) 8:e54932. doi: 10.1371/journal.pone.0054932. PMID: 23383003 PMC3557299

[174] Duong FH Christen V Lin S Heim MH . Hepatitis C virus-induced up-regulation of protein phosphatase 2A inhibits histone modification and DNA damage repair. Hepatology. (2010) 51:741–51. doi: 10.1002/hep.23388. PMID: 20043320

[175] Tarhini AA Lin Y Yeku O LaFramboise WA Ashraf M Sander C . A four-marker signature of TNF-RII, TGF-alpha, TIMP-1 and CRP is prognostic of worse survival in high-risk surgically resected melanoma. J Transl Med. (2014) 12:19. doi: 10.1186/1479-5876-12-19. PMID: 24457057 PMC3909384

[176] Ricca TI Liang G Suenaga AP Han SW Jones PA Jasiulionis MG . Tissue inhibitor of metalloproteinase 1 expression associated with gene demethylation confers anoikis resistance in early phases of melanocyte Malignant transformation. Transl Oncol. (2009) 2:329–40. doi: 10.1593/tlo.09220. PMID: 19956395 PMC2781071

[177] Wilting SM Miok V Jaspers A Boon D Sorgard H Lando M . Aberrant methylation-mediated silencing of microRNAs contributes to HPV-induced anchorage independence. Oncotarget. (2016) 7:43805–19. doi: 10.18632/oncotarget.9698. PMID: 27270309 PMC5190061

[178] Wilting SM van Boerdonk RA Henken FE Meijer CJ Diosdado B Meijer GA . Methylation-mediated silencing and tumour suppressive function of hsa-miR-124 in cervical cancer. Mol Cancer. (2010) 9:167. doi: 10.1186/1476-4598-9-167. PMID: 20579385 PMC2917428

[179] Castaneda M den Hollander P Kuburich NA Rosen JM Mani SA . Mechanisms of cancer metastasis. Semin Cancer Biol. (2022) 87:17–31. doi: 10.1016/j.semcancer.2022.10.006. PMID: 36354098

[180] Kolbl AC Jeschke U Andergassen U . The significance of epithelial-to-mesenchymal transition for circulating tumor cells. Int J Mol Sci. (2016) 17(8):1308. 27529216 10.3390/ijms17081308 PMC5000705

[181] Neophytou CM Kyriakou TC Papageorgis P . Mechanisms of metastatic tumor dormancy and implications for cancer therapy. Int J Mol Sci. (2019) 20(24):6158. doi: 10.3390/ijms20246158. PMID: 31817646 PMC6940943

[182] Wang J Li D Cang H Guo B . Crosstalk between cancer and immune cells: Role of tumor-associated macrophages in the tumor microenvironment. Cancer Med. (2019) 8:4709–21. doi: 10.1002/cam4.2327. PMID: 31222971 PMC6712467

[183] Beerling E Seinstra D de Wit E Kester L van der Velden D Maynard C . Plasticity between epithelial and mesenchymal states unlinks EMT from metastasis-enhancing stem cell capacity. Cell Rep. (2016) 14:2281–8. doi: 10.1016/j.celrep.2016.02.034. PMID: 26947068 PMC4802222

[184] Chaffer CL Brennan JP Slavin JL Blick T Thompson EW Williams ED . Mesenchymal-to-epithelial transition facilitates bladder cancer metastasis: role of fibroblast growth factor receptor-2. Cancer Res. (2006) 66:11271–8. doi: 10.1158/0008-5472.can-06-2044. PMID: 17145872

[185] Lu W Kang Y . Epithelial-mesenchymal plasticity in cancer progression and metastasis. Dev Cell. (2019) 49:361–74. doi: 10.1016/j.devcel.2019.04.010. PMID: 31063755 PMC6506183

[186] Ruscetti M Quach B Dadashian EL Mulholland DJ Wu H . Tracking and functional characterization of epithelial-mesenchymal transition and mesenchymal tumor cells during prostate cancer metastasis. Cancer Res. (2015) 75:2749–59. doi: 10.1158/0008-5472.can-14-3476. PMID: 25948589 PMC4490048

[187] Taube JH Sphyris N Johnson KS Reisenauer KN Nesbit TA Joseph R . The H3K27me3-demethylase KDM6A is suppressed in breast cancer stem-like cells, and enables the resolution of bivalency during the mesenchymal-epithelial transition. Oncotarget. (2017) 8:65548–65. doi: 10.18632/oncotarget.19214. PMID: 29029452 PMC5630352

[188] Udali S De Santis D Ruzzenente A Moruzzi S Mazzi F Beschin G . DNA methylation and hydroxymethylation in primary colon cancer and synchronous hepatic metastasis. Front Genet. (2017) 8:229. doi: 10.3389/fgene.2017.00229. PMID: 29375619 PMC5767180

[189] Pfeifer GP Xiong W Hahn MA Jin SG . The role of 5-hydroxymethylcytosine in human cancer. Cell Tissue Res. (2014) 356:631–41. doi: 10.1007/s00441-014-1896-7. PMID: 24816989 PMC4090033

[190] Tong M Gao S Qi W Shi C Qiu M Yang F . 5-hydroxymethylcytosine as a potential epigenetic biomarker in papillary thyroid carcinoma. Oncol Lett. (2019) 18:2304–9. doi: 10.3892/ol.2019.10531. PMID: 31452729 PMC6676597

[191] Barciszewska AM . Global DNA demethylation as an epigenetic marker of human brain metastases. Biosci Rep. (2018) 38(5):BSR20180731. doi: 10.1042/bsr20180731. PMID: 30254100 PMC6200709

[192] Estevao-Pereira H Lobo J Salta S Amorim M Lopes P Cantante M . Overexpression of circulating MiR-30b-5p identifies advanced breast cancer. J Transl Med. (2019) 17:435. doi: 10.1186/s12967-019-02193-y. PMID: 31888645 PMC6936051

[193] Feng YH Tsao CJ . Emerging role of microRNA-21 in cancer. BioMed Rep. (2016) 5:395–402. doi: 10.3892/br.2016.747. PMID: 27699004 PMC5038362

[194] Sahay D Leblanc R Grunewald TG Ambatipudi S Ribeiro J Clezardin P . The LPA1/ZEB1/miR-21-activation pathway regulates metastasis in basal breast cancer. Oncotarget. (2015) 6:20604–20. doi: 10.18632/oncotarget.3774. PMID: 26098771 PMC4653029

[195] Wang C Danli M Yu H Zhuo Z Ye Z . N6-methyladenosine (m6A) as a regulator of carcinogenesis and drug resistance by targeting epithelial-mesenchymal transition and cancer stem cells. Heliyon. (2023) 9:e14001. doi: 10.1016/j.heliyon.2023.e14001. PMID: 36915498 PMC10006539

[196] Zhao X Li X Li X . Multiple roles of m(6)A methylation in epithelial-mesenchymal transition. Mol Biol Rep. (2022) 49:8895–906. doi: 10.1007/s11033-022-07368-3. PMID: 35306622

[197] Zhou Y Cao P Zhu Q . The regulatory role of m6A in cancer metastasis. Front Cell Dev Biol. (2025) 13:1539678. doi: 10.3389/fcell.2025.1539678. PMID: 40356596 PMC12066624

[198] Ru W Zhang X Yue B Qi A Shen X Huang Y . Insight into m(6)A methylation from occurrence to functions. Open Biol. (2020) 10:200091. doi: 10.1098/rsob.200091. PMID: 32898471 PMC7536083

[199] Li J Xie G Tian Y Li W Wu Y Chen F . RNA m(6)A methylation regulates dissemination of cancer cells by modulating expression and membrane localization of beta-catenin. Mol Ther. (2022) 30:1578–96. doi: 10.1016/j.ymthe.2022.01.019. PMID: 35033632 PMC9077323

[200] Lin X Chai G Wu Y Li J Chen F Liu J . RNA m(6)A methylation regulates the epithelial mesenchymal transition of cancer cells and translation of Snail. Nat Commun. (2019) 10:2065. doi: 10.1038/s41467-023-43307-x. PMID: 31061416 PMC6502834

[201] Wanna-Udom S Terashima M Lyu H Ishimura A Takino T Sakari M . The m6A methyltransferase METTL3 contributes to transforming growth factor-beta-induced epithelial-mesenchymal transition of lung cancer cells through the regulation of JUNB. Biochem Biophys Res Commun. (2020) 524:150–5. doi: 10.1016/j.bbrc.2020.01.042. PMID: 31982139

[202] Yue B Song C Yang L Cui R Cheng X Zhang Z . METTL3-mediated N6-methyladenosine modification is critical for epithelial-mesenchymal transition and metastasis of gastric cancer. Mol Cancer. (2019) 18:142. doi: 10.1186/s12943-019-1065-4. PMID: 31607270 PMC6790244

[203] Zhao C Ling X Xia Y Yan B Guan Q . The m6A methyltransferase METTL3 controls epithelial-mesenchymal transition, migration and invasion of breast cancer through the MALAT1/miR-26b/HMGA2 axis. Cancer Cell Int. (2021) 21:441. doi: 10.1186/s12935-021-02113-5. PMID: 34419065 PMC8380348

[204] Li D Xu L Liu R Yao Z Zheng C Jin S . MAZ-mediated N6-methyladenosine modification of ZEB1 promotes hepatocellular carcinoma progression by regulating METTL3. J Transl Med. (2025) 23:265. doi: 10.1186/s12967-025-06314-8. PMID: 40038747 PMC11877864

[205] Sun Z Su Z Zhou Z Wang S Wang Z Tong X . RNA demethylase ALKBH5 inhibits TGF-beta-induced EMT by regulating TGF-beta/SMAD signaling in non-small cell lung cancer. FASEB J. (2022) 36:e22283. doi: 10.1096/fj.202200005rr. PMID: 35344216

[206] Jin D Guo J Wu Y Yang L Wang X Du J . m(6)A demethylase ALKBH5 inhibits tumor growth and metastasis by reducing YTHDFs-mediated YAP expression and inhibiting miR-107/LATS2-mediated YAP activity in NSCLC. Mol Cancer. (2020) 19:40. doi: 10.1186/s12943-020-01161-1. PMID: 32106857 PMC7045432

[207] Hu W Klumper N Schmidt D Ritter M Ellinger J Hauser S . Depletion of the m(6)A demethylases FTO and ALKBH5 impairs growth and metastatic capacity through EMT phenotype change in clear cell renal cell carcinoma. Am J Transl Res. (2023) 15:1744–55. doi: 10.1097/rlu.0000000000006385. PMID: 37056835 PMC10086911

[208] Shen Q Deng E Luo L Zhang J Yang Q Su D . High-throughput single-cell DNA methylation and chromatin accessibility co-profiling with SpliCOOL-seq. Clin Transl Med. (2026) 16:e70584. doi: 10.1101/2025.02.25.640234. PMID: 41603298 PMC12848781

[209] Ma L Guo H Zhao Y Liu Z Wang C Bu J . Liquid biopsy in cancer current: status, challenges and future prospects. Signal Transduct Target Ther. (2024) 9:336. doi: 10.1038/s41392-024-02021-w. PMID: 39617822 PMC11609310

[210] Song J Yang P Chen C Ding W Tillement O Bai H . Targeting epigenetic regulators as a promising avenue to overcome cancer therapy resistance. Signal Transduct Target Ther. (2025) 10:219. doi: 10.1038/s41392-025-02266-z. PMID: 40675967 PMC12271501

[211] Dai W Qiao X Fang Y Guo R Bai P Liu S . Epigenetics-targeted drugs: current paradigms and future challenges. Signal Transduct Target Ther. (2024) 9:332. doi: 10.1038/s41392-024-02039-0. PMID: 39592582 PMC11627502

[212] Sadida HQ Abdulla A Marzooqi SA Hashem S Macha MA Akil ASA . Epigenetic modifications: Key players in cancer heterogeneity and drug resistance. Transl Oncol. (2024) 39:101821. doi: 10.1016/j.tranon.2023.101821. PMID: 37931371 PMC10654239

[213] Ma C Cheng J Gu J Wang Q . Epigenetic drugs in cancer therapy: mechanisms, immune modulation, and therapeutic applications. Mol BioMed. (2025) 6:132. doi: 10.1186/s43556-025-00373-5. PMID: 41335282 PMC12675902

[214] Cavallari I Ciccarese F Sharova E Urso L Raimondi V Silic-Benussi M . The miR-200 family of microRNAs: Fine tuners of epithelial-mesenchymal transition and circulating cancer biomarkers. Cancers (Basel). (2021) 13(23):5874. doi: 10.3390/cancers13235874. PMID: 34884985 PMC8656820

[215] Zhu Y Liu K Zhu H . Targeting epigenetic methylation: emerging diagnosis and therapeutic strategies in cancer. Exp Hematol Oncol. (2026) 15:27. doi: 10.1186/s40164-026-00760-w. PMID: 41673722 PMC12922360

[216] Pandey S Yadav P . Liquid biopsy in cancer management: Integrating diagnostics and clinical applications. Pract Lab Med. (2025) 43:e00446. doi: 10.1016/j.plabm.2024.e00446. PMID: 39839814 PMC11743551

[217] Nowak E Bednarek I . Aspects of the epigenetic regulation of EMT related to cancer metastasis. Cells. (2021) 10(12):3435. doi: 10.3390/cells10123435. PMID: 34943943 PMC8700111

[218] Prasetyanti PR Medema JP . Intra-tumor heterogeneity from a cancer stem cell perspective. Mol Cancer. (2017) 16:41. doi: 10.1186/s12943-017-0600-4. PMID: 28209166 PMC5314464

